# Who Bites the Bullet First? The Susceptibility of Leopards *Panthera pardus* to Trophy Hunting

**DOI:** 10.1371/journal.pone.0123100

**Published:** 2015-04-10

**Authors:** Alex Richard Braczkowski, Guy Andrew Balme, Amy Dickman, David Whyte Macdonald, Julien Fattebert, Tristan Dickerson, Paul Johnson, Luke Hunter

**Affiliations:** 1 Wildlife Conservation Research Unit, Department of Zoology, University of Oxford, Recanati-Kaplan Centre, Tubney House, Tubney, Oxford, United Kingdom; 2 Panthera, New York, New York, United States of America; 3 Department of Biological Sciences, University of Cape Town, Rondebosch, South Africa; 4 Ruaha Carnivore Project, Iringa, Tanzania; 5 School of Life Sciences, University of Kwazulu-Natal, Durban, South Africa; Instituto de Higiene e Medicina Tropical, PORTUGAL

## Abstract

Reliable data is fundamentally important for managing large carnivore populations, and vital for informing hunting quota levels if those populations are subject to trophy hunting. Camera-trapping and spoor counts can provide reliable population estimates for many carnivores, but governments typically lack the resources to implement such surveys over the spatial scales required to inform robust quota setting. It may therefore be prudent to shift focus away from estimating population size and instead focus on monitoring population trend. In this paper we assess the susceptibility of African leopards *Panthera pardus* to trophy hunting. This has management ramifications, particularly if the use of harvest composition is to be explored as a metric of population trend. We explore the susceptibility of different leopard age and sex cohorts to trophy hunting; first by examining their intrinsic susceptibility to encountering trophy hunters using camera-traps as surrogates, and second by assessing their extrinsic susceptibility using photographic questionnaire surveys to determine their attractiveness to hunters. We show that adult male and female leopards share similar incident rates to encountering hunters but adult males are the most susceptible to hunting due to hunter preference for large trophies. In contrast, sub-adult leopards rarely encounter hunters and are the least attractive trophies. We suggest that our findings be used as a foundation for the exploration of a harvest composition scheme in the Kwazulu-Natal and Limpopo provinces where post mortem information is collected from hunted leopards and submitted to the local provincial authorities.

## Introduction

Trophy hunting is a popular wildlife management tool that has potential to contribute to species’ conservation [1, 2]. It can generate important revenue for landowners, contribute to national Gross Domestic Products (GDP), and hunters may enforce anti-poaching and land management approaches that protect wildlife and natural habitat [1]. Trophy hunting differs from other forms of harvest (e.g. for bushmeat or the traditional medicinal trade) in that offtake can be regulated and is typically selective, focusing on individuals with attractive secondary sexual attributes such as large horns, tusks or manes [3–5]. However, when poorly managed (e.g. when young animals are targeted or quotas are too high), trophy hunting can cause social disruption [6], the inheritance of undesirable traits [7] and localized population declines [8,2].

Ideally, trophy hunting quotas should be based on robust population estimates, but wildlife management authorities rarely possess such data because of time, funding and logistical constraints [9]. Accurately estimating population size is particularly challenging for wide-ranging, cryptic species such as large carnivores, which are also those most sensitive to the effects of trophy hunting [10–12]. Often the only data available are post-mortem reports that include the effort and success of hunts, and the sex and estimated age of harvested animals [13], but these age-sex ratios of harvested individuals can provide a useful index of population trend [14, 12]. However, an important caveat of such an approach is that relative susceptibility to hunting varies predictably among age and sex classes. Several factors may influence susceptibility to hunting, most notably the movement patterns of individual animals (‘intrinsic susceptibility’) [12, 15] and their attractiveness to hunters (‘extrinsic susceptibility’) [16]). Susceptibility could also be influenced by the configuration of home-ranges and their consequent accessibility by hunters. We use a combination of camera-trapping, radio-tracking and interview data to assess both the intrinsic and extrinsic susceptibility to hunting of different age and sex classes of African leopards.

Leopards are one of the most sought-after big game trophies; presently, 12 African countries are permitted by the Convention for the International Trade in Endangered Species (CITES) to export a collective 2648 leopard skins (Table 1) procured through trophy hunting each year [17]. Due to a widespread paucity of data on leopard numbers [18], most range states base leopard hunting quotas on expert guesstimates or an over-simplified model that correlated leopard density to rainfall [19] but ignored important factors such as anthropogenic mortality and prey availability [20]. This is of particular concern as leopards are demographically sensitive to over-harvesting, and an increased turnover of adult males may cause inflated rates of infanticide, which are already naturally high in leopard populations [21]. Moreover, leopards are important revenue generators for both photo safari operators [22] and hunters, with leopards contributing 8–20% of gross national trophy hunting income in East and southern Africa [1]. Many African countries already mandate trophy hunters to provide post-hunt data, which include photographic, morphometric and dental information that can be used for accurate ageing and sexing of harvested individuals [23]. We propose that with our estimates of susceptibility to hunting, this would be an important first step to explore the use of harvest composition as an index of leopard population trend. This is a method that has been used with success for the Puma *Puma concolor* in Wyoming, and we suggest a similar trial in Africa for leopard, a data deficient species across much of its range. As such, harvest composition has the potential to act as a valuable tool for monitoring leopard populations at meaningful management scales to inform conservation decisions.

**Table 1:** Country size, CITES quota size and mean trophy exports for the 2006–2010 period obtained from the CITES database.

Country	Country size (km^2^)	Quota	5 year mean export
Botswana	600 370	130	44 ± 6.69
Central African Republic	622 984	40	23.6 ± 6.67
Ethiopia^£^	1 127 127	500	0^£^
Kenya^$^	582 650	80	0^$^
Malawi*	118 480	50	0*
Mozambique	801 590	120	37 ± 2.81
Namibia	825 418	250	197.4 ± 32.46
South Africa	1 219 912	150	114.2 ± 11.73
Tanzania	945 203	500	280.4 ± 28.18
Zambia	752 614	300	68.8 ± 6.21
Zimbabwe	390 580	500	251.4 ± 11.72
Uganda	236 040	28	1 ± 1

£ Ethiopia has no records of exports/imports in the CITES database and at present leopards are not hunted there (Hans Bauer, personal.communication).

$ Kenya allow the export of leopard body parts but trophy hunting was outlawed in 1977.

*There were no leopard export records found in the CITES database for the 2006–2010 period in Malawi.

## Methods

### Ethics Statement

The GPS collar data used in this paper originates in part from previous research implemented by [24]. The original permission to radio-collar leopards on Phinda Game Reserve was granted by the provincial conservation authority, Ezemvelo Kwazulu-Natal Wildlife (permit number HO/4004/07), as well as by & Beyond, the reserve management on Phinda Game Reserve. Ethical clearance for collaring was also provided by the University of Kwazulu-Natal Ethics Committee (approval 051/12/Animal). We also examined Oxford University’s Central University Research Ethics Committee’s (CERU) ethics approval checklist in order to gauge whether our photographic survey work required further ethical audit. According to this checklist our work does not require further ethical audit. Specifically, the names of participants involved in our photographic survey were coded and identifiers removed. Participants were also informed that they would be anonymous and that the results emanating from the survey would be published in a peer-reviewed journal.

### Study Area

**Fig 1:**
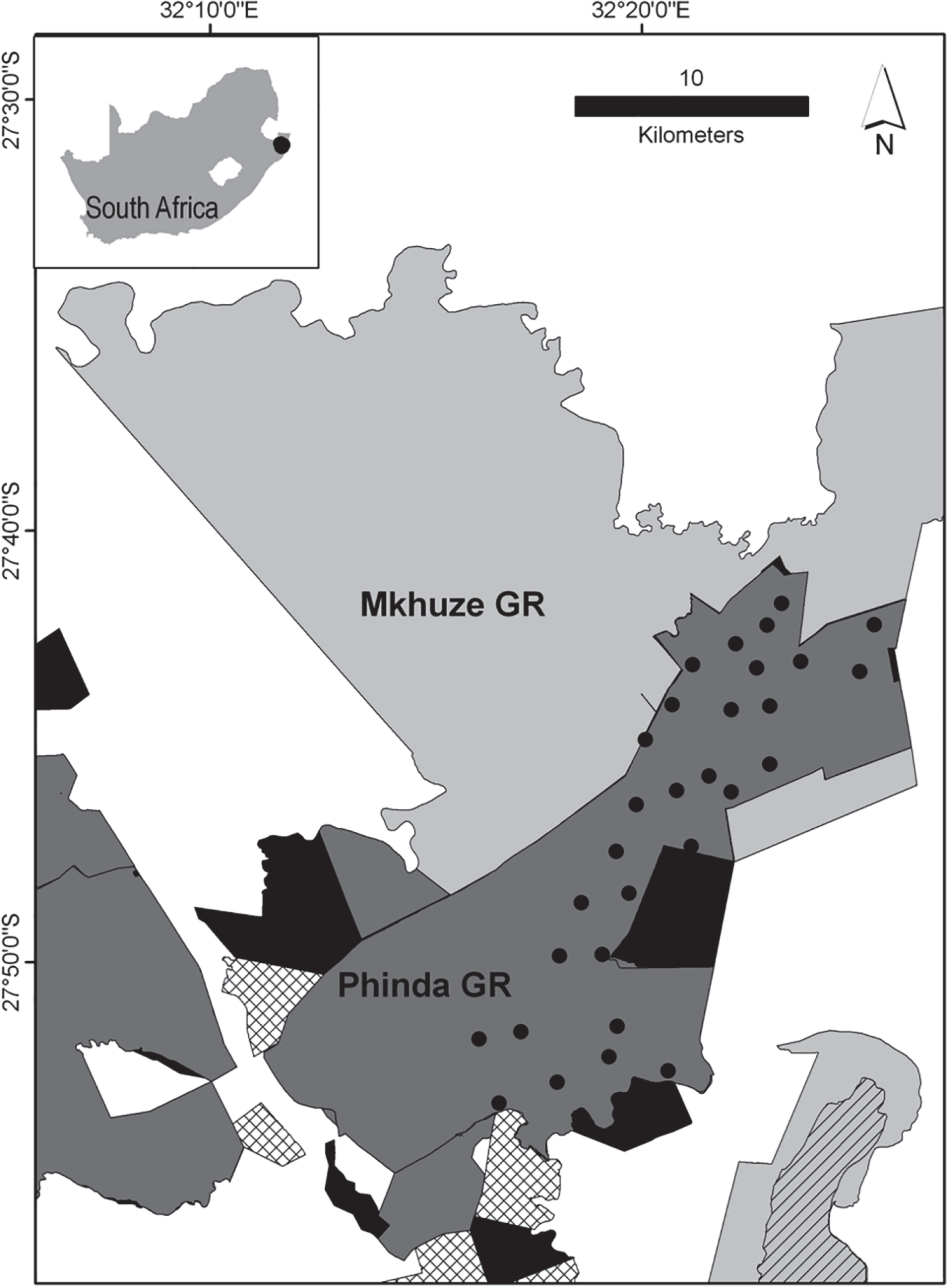
The location of the Phinda Private Game Reserve (Dark grey) with camera-trap stations (black) with the adjacent Mkhuze game reserve (light grey). Private game ranches (black) and cattle farms (hatched) are also provided for reference.

We collected data on leopard population dynamics in Phinda Private Game Reserve (27^0^ 51’ 30” S, 32^0^ 19’ 00” E, hereafter Phinda) between April 2002 and December 2012. Phinda forms part of the Munyawana Conservancy (270km^2^) which is located in the Maputaland-Pondoland-Albany Biodiversity Hotspot in northern KwaZulu-Natal South Africa (Fig 1). Phinda receives an average of 550 mm of rainfall annually, which falls mainly between October and March. Phinda’s vegetation is dominated by several varieties of savanna, but broad-leafed woodland interspersed with grassland is the most common physiognomic form [24]. Forty-two large mammal species have been recorded on Phinda, including important leopard prey such as nyala *Tragelaphus angasii*, impala *Aepyceros melampus* and warthog *Phacochoerus africanus* [24]. The data used in this study originate from a long-term study on the species. The leopards on Phinda have experienced varied levels of persecution as attributed to trophy hunting and we feel it therefore comprises a useful study population for the exploration of hunting susceptibility.

### Likelihood of a leopard encountering a hunter (intrinsic susceptibility)

Leopards are usually hunted from a stationary hide or blind positioned 50–80 m from a bait (typically the carcass of a locally common prey species) hung in a tree [25]. We used camera-trap data of known collared individuals to estimate the relative frequency that different age and sex classes of leopards encounter blinds. Hunters typically drag baits along game trails or dirt roads for 500–1000 m to lure an animal to a blind [25]. The likelihood of an individual responding to a lure may vary in a population [26], but we demonstrated that response rates (as determined by photographic captures) were similar among leopard age and sex classes at our study site [27]. We were therefore confident that the absence of a bait at camera-trap stations was unlikely to affect our cohort-specific encounter rates.

We undertook four 40-day camera-trap surveys in 2005, 2007, 2009 and 2011. All surveys took place during the dry season. We deployed 30 paired stations, comprising 35-mm Deercam Dc300 (Deercam, Park Falls, WI, USA) and Panthera IV digital camera-traps, across 140 km^2^ [28]. Hunters usually set 3–5 baits during a leopard hunt and the mean size of property on which leopard hunts are conducted in KwaZulu-Natal is roughly 25 km^2^ (Ezemvelo KwaZulu-Natal Wildlife, unpublished data). Hence, the density of camera-traps in our study area was comparable to the density and spacing of baits deployed during a typical leopard hunt. The distribution of camera-traps also ensured that each leopard was capable of being captured (i.e. ≥4 stations were present in the mean female leopard home-range recorded on Phinda; mean = 29.4±1.5 km^2^; [29]). Camera-traps were checked every 2–3 days to replace batteries and film, and to download images. Individual leopards were distinguished using their unique pelage patterns [30] and assigned to one of three demographic cohorts based on their morphological characteristics (presence of testes in males, dewlap development, facial scarring and dental wear) [23]: 1) adult males≥3 years; 2) adult females ≥3 years, and 3) or sub-adults of 1–3 years (transients), (Table 2). We grouped male and female sub-adults due to small samples sizes, and because we found no difference in the mean numbers of photographs recorded between sub-adult sexes (Two-sample t-test; d.f = 1; t = 0.88; p = 0.54). If a leopard changed cohort from one survey to the next (i.e. a sub-adult became an adult), it was re-assigned to the appropriate cohort [31].

**Table 2:** Number of daily telemetry locations, photographic events and the relative time spent in the study area by radio-collared leopards in Phinda Private Game Reserve, South Africa (2005–2011).

Year	Cohort	Collared individuals	Total number of telemetry locations	Telemetry locations inside study area	Mean relative time inside study area	Number of captures (collared population)	Number of captures (total population)
2005	♂	4	217	157	72.35	14	16
	♀	5	175	166	94.86	18	21
	Subs	2	55	52	94.55	2	2
2007	♂	1	14	14	100.00	2	10
	♀	4	138	120	86.96	11	12
	Subs	6	114	77	67.54	12	16
2009	♂	0	NA	NA	NA	0	27
	♀	6	96	88	91.67	20	21
	Subs	2	21	20	95.24	1	5
2011	♂	2	179	144	80.45	8	24
	♀	3	164	158	96.34	6	23
	Subs	4	139	90	64.75	2	8
**Total**		**39**	**1312**	**1086**	**84.20**	**96**	**185**

For each of the four camera-trap surveys, we investigated how many times each member of a cohort encountered a camera-trap station, correcting for the proportion of time they spent in the survey area (Table 2). A sample of leopards was radio-collared throughout the study (see [24] for full details of capture and immobilization); hence, we used radio-telemetry data to estimate the proportion of time leopards spent in the survey area. In order to provide sufficient spatial data, we present three months of radio-telemetry data, with each 40-day camera-trap survey in the middle of the three months. We used only one location per individual per day to ensure data from VHF and GPS collared leopards were comparable [32]. We added a buffer equal to the mean camera-trap spacing (1.67 km) to the outermost camera-traps, and divided the number of telemetry locations recorded for each leopard inside the buffered survey area by the total number of telemetry locations collected over the three-month radio-tracking period.

We used a General Linear Model (GLM) to assess cohort-specific encounter rates to camera-traps. We examined the effects that cohort, year and the relative time spent by individual leopards in the survey area had on the total number of captures recorded for each collared leopard. The relative time each leopard spent within the survey area was used as the offset parameter in the model and we censored any animal with <10 telemetry locations for a given 3-month period. To test the robustness of using a minimum threshold of ≥10 locations for each leopard, we created a second model using only animals with ≥30 locations and compared those parameter estimates to the original model. Models were fitted with a negative binomial distribution using the MASS package in R as this provides a better fit for over-dispersed count data when compared to the Poisson distribution [33]. We created four candidate models incorporating cohort, year and offset parameters and assessed them according to Aikaike’s Information Criterion [33].

We hypothesized that adult male leopards would be recorded on camera-traps with a higher frequency than adult females and sub-adults due to (a) males ranging more widely [34, 35] and (b) potential avoidance of trails and roads by females, which are frequently patrolled by adult males [36, 37]. We also hypothesized that due to their use of temporary home ranges [38], sub-adult leopards would be photographed at a lower rate than adults.

### Attractiveness of leopards to hunters (extrinsic susceptibility)

We examined the relative attractiveness of leopard cohorts to trophy hunters using a structured questionnaire survey. Survey participants (professional hunters—PH) were randomly selected from the membership lists of national professional hunting associations from the main leopard hunting countries [23]. The questionnaire contained 25 side-profile photographs (minimum 300 dpi) of different known-age and sex leopards from a long-term study in the Sabi Sand Game Reserve, South Africa (S1 Fig). Hunters were asked whether they would choose to shoot a leopard, and on which day during a 14-day hunting safari they would make this decision (14 days is the typical duration for leopard hunts in East and southern Africa; [39]). Responses were placed on an ordinal scale (a) 3 = willing to shoot on the first day of a 14-day safari; (b) 2 = willing to shoot on the seventh day of a 14-day safari; (c) 1 = willing to shoot on the fourteenth day of a 14-day safari (d) 0 = unwilling to shoot the leopard at any stage of the hunt. Scores therefore reflected relative trophy preference, with leopards scoring closer to three more attractive to hunters than those closer to zero. We used the same demographic cohorts used in the camera-trap analysis to build two GLMs: 1) the first examined leopard attributes, i.e. the effects of leopard age and sex on the average score that each photograph achieved. The scores averaged across respondents were treated as continuous. A normal errors model was used; inspection of diagnostic plots indicated no deviation from the assumption of normality and homogeneity of residuals. 2) The second explored GLM the effect of hunter attributes, i.e. the effects of hunter experience, in terms of the number of years that the PH had guided hunts, the number of leopard hunts they had guided, and the number of countries in which they had hunted leopards. For this analysis the average score assigned to photographs across the sample of hunters was used as the response, also using a normal errors model.

We hypothesized that photographs of adult male leopards would be rated more highly higher than those of adult female and sub-adults due to their larger body size [23]. We also hypothesized that more experienced hunters would exercise greater caution in deciding to shoot animals, and therefore record lower scores than less experienced hunters.

All statistical analyses were performed in the R statistical environment [40] and results are provided with means ($\bar{x}$ ± S.E) and standard error as a measure of precision.

## Results

### Intrinsic susceptibility

On average, we obtained 19.3±3.9 photographs of adult male leopards, 19.3±0.1 of adult female leopards, and 7.8±2.6 of subadults during each of the four surveys (Table 1). Of these, 35±17%, 75±37%, and 55±28% were of collared individuals respectively. We used 119±7 telemetry locations (range = 14–217) from 9.8±0.7 individuals (range = 8–11) per survey to construct our GLM. We compared four possible candidate models, and used a model which omitted the influence of year as it was not a significant predictor of captures, and yielded a lower AIC score (AIC = 155.63 compared to AIC = 160.31; Table 3) compared to a model with a year effect. Exponentiation of our GLM coefficients indicated that adult male (exponentiated estimate = 3.27 (95% lower = 1.40; 95% upper = 11.30)) and adult female leopards (exponentiated estimate = 4.00 (95% lower = 2.26; 95% upper = 4.69)) were similarly likely to encounter a camera-trap, when accounting for the proportion of time they spent in the survey area (Table 4). Sub-adults were less likely to encounter a camera-trap (exponentiated estimate = 1.64; 95% lower = 0.57; 95% upper = 4.55)) than were either adult males or adult females. Our GLM including only animals with ≥30 telemetry locations performed similarly to the original model, except for sub-adult parameter estimates, which were near significant (S1 Table).

**Table 3:** Four candidate models with the number of leopard photographic events as the response variable evaluated using AIC criteria.

Model parameter	Parameters	DF	AIC
Events ~ Cohort + Offset	2	4	155.63
Events ~ Cohort + Year + Offset	3	7	160.31
Events ~ Cohort	1	5	162.10
Events ~ Year + Offset	2	4	162.31

**Table 4:** Parameter estimates from a General Linear Model (GLM) examining the intrinsic susceptibility of leopard cohorts to trophy hunting, measured by encounter rates of leopards to camera-traps, which are used as surrogates for hunters.

Coefficients	Estimate	2.5% CI	97.5% CI	Exponentiated estimate	Std.error	z-value	Pr (>|z|)
Intercept (Adult females)	-3.42	-3.79	-3.06	3.27	0.19	-18.36	<0.005
Adult males	0.2	-0.48	0.87	4.00	0.35	0.58	0.56
Sub-adults	-0.69	-1.38	-0.03	1.64	0.34	-2.01	0.04

Residual deviance: 46.24 on 36 d.f

AIC: 155.63

Theta: 3.42

### Extrinsic susceptibility

The questionnaire survey was completed by 39 professional hunters that had hunted leopards in Botswana, South Africa, Namibia, Tanzania, Zambia and Zimbabwe (there were no missing responses). Our GLM assessing the relative attractiveness of cohorts to hunters suggested that adult males were the most attractive cohort to hunters (exponentiated estimate = 14.30; 95% lower = 3.86; 95% upper = 52.98))), followed by females (exponentiated estimate = 3.71; 95% lower = 2,05; 95% upper = 6,75)) while sub-adults were least attractive (exponentiated estimate = 2.44; 95% lower = 0.64; 95% upper = 9.30; Table 5). Adult males (mean = 2.66±0.12) scored significantly higher than adult females (mean = 1.31±0.30) and sub-adults (mean = 0.90±0.17; Tukey test p = <0.05).

**Table 5:** Parameter estimates from a General Linear Model (GLM) examining the extrinsic susceptibility of leopard cohorts to trophy hunting, derived from scores of the willingness of hunters to shoot leopards presented in photographic questionnaire survey.

Coefficients	Estimate	2.5% CI	97.5% CI	Exponentiated estimate	Std.error	t-value	Pr (>|z|)
Intercept (Adult females)	1.31	0.72	1.91	3.71	0.29	4.6	<0.005
Adult males	1.35	0.63	2.06	14.30	0.34	3.91	<0.005
Sub-adults	-0.42	-1.16	0.32	2.44	0.36	-1.17	0.25

Residual deviance: 8.96 on 22 d.f

Our GLM examining hunter experience revealed that hunters who had hunted for longer scored significantly lower than less experienced hunters (F_(1,33)_ = 9.18, p = <0.005). Similarly hunters which guided more hunts scored lower than those with fewer hunts (F_(2,33)_ = 3.73, p = 0.05). There was little evidence that the number of countries hunted in affected the scores (F_(2,33)_ = 0.11; p = 0.90).

## Discussion

Our results support the hypothesis that leopards exhibit varying susceptibility to trophy hunting. They suggest that adult male and female leopards share a similar risk of encountering a trophy hunter, if we can assume that the likelihood of encountering a camera is a useful surrogate, but that adult males are more attractive as trophies and are thus more susceptible to harvesting. Sub-adult leopards are the least susceptible cohort as they would seldom encounter a hunter and, even if encountered, are less attractive as trophies. Our findings differ from susceptibility indices estimated for puma *Puma concolor*, which suggest transient males are the most susceptible to harvesting [10]. This highlights the need for species-specific susceptibility estimates, which take into account all factors likely to influence the relative vulnerability of different age and sex classes (e.g. the biology of the target species, harvest methods, etc.). Interestingly, we did not record a marked male-bias in capture rates as documented by other camera-trap studies on leopard [36, 37] and jaguar *Panthera onca* [41, 42]. We attribute this to the concentration of camera-traps in our survey area, which appear to have captured the movements of adult females satisfactorily [28].

The lower encounter rates recorded for sub-adults are likely due to their spatial patterns. Dispersing sub-adult carnivores (including leopard; [43]) often establish small, temporary home ranges in which they settle for a few months before making a considerable foray. For example [44] found that dispersing Californian pumas occupied home ranges as small as 2% of the size of adult male ranges, sometimes for several months. Such confined movement would strongly reduce the probability of a sub-adult encountering a camera-trap. Exploratory forays by dispersing sub-adults following the abandonment of a temporary home range also make them prone to using areas outside the area sampled by cameras [29].

The relative attractiveness of leopards as trophies appears to be solely a function of their size. Leopards exhibit striking sexual size dimorphism, with adult males typically 60% larger than females [23, 45] and it was thus unsurprising that adult males were preferred by hunters. Adult females and sub-adults, in contrast, are similarly sized [23] and hunters showed no preference for either class. Our results support the findings of [23] which suggested hunters could distinguish mature males but not females and sub-adults. Worryingly, many hunters (87% of respondents) report willingness to hunt a female at some stage during a hunt, even though this is illegal in most countries [17]. Using genetic data, [46] similarly showed that females comprised 27% of 77 leopard trophies hunted in Tanzania between 1995 and 1998, even though only males can be hunted there legally. The hunting of adult females has important ramifications for population viability as they are the key reproductive unit [47] and are typically more difficult to replace than adult males, due to male-biased dispersal [10]. Stipulating (and strictly enforcing) that only mature, adult males can be hunted will essentially eliminate the possibility of hunters mistakenly harvesting females. Restricting offtakes to males aged ≥7 years would further improve the sustainability of trophy hunting, as by this age male leopards have had the opportunity to rear at least one litter to independence which is sufficient to ensure population persistence [8]. We acknowledge that susceptibility is also likely to be affected by a measure of catch per unit effort over a typical 14 day hunt. A professional hunting guide is therefore also likely to influence his/her client, based upon previous experience of how many leopards they saw over previous hunts.

## Conclusions

The sustainable management of leopard hunting has previously been hampered by a lack of reliable population data and, as a consequence, quotas are typically set in an arbitrary fashion, sometimes leading to population declines [17].Our findings suggest that African leopards exhibit varying levels of susceptibility to trophy hunting. We suggest that harvest composition may therefore have potential as an index of leopard population trend, especially as the most susceptible cohorts are likely to be removed from populations first (this indeed was the case with puma in Wyoming [10]). In our case, a proportional increase in adult female and sub-adult in the harvest composition would suggest a declining or over-harvest population since the most vulnerable cohort (adult male) should be depleted first. Thus, wildlife agencies can use this to facilitate the adaptive management of leopard hunting, adjusting quotas based on the sex and age of individuals harvested. Similar approaches have been applied successfully in the past to manage the trophy hunting of lions *Panthera leo* [48] and pumas [10]. For leopards, detailed photographs showing the relative body dimensions and tooth wear (S2 Fig) can be used to age trophies [23], and a small tissue sample collected to molecularly validate gender [49]. It requires that hunters submit accurate and complete data for every leopard harvested, but this can be enforced by authorities using penalties such as a reduction in future quotas or even the confiscation of trophies.

## Supporting Information

S1 Fig Questionnaire survey used to examine the extrinsic susceptibility of leopards to trophy hunting (i.e. their relative attractiveness to professional hunters). Respondents were asked whether they would choose to shoot a leopard, and on which day during a 14-day hunting safari they would make this decision: (a) 3 = willing to shoot on the first day of a 14-day safari; (b) 2 = willing to shoot on the seventh day of a 14-day safari; (c) 1 = willing to shoot on the fourteenth day of a 14-day safari (d) 0 = unwilling to shoot the leopard at any stage of the hunt. All photographs are of known sex and age leopards from the Sabi Sand Game Reserve, South Africa.
(PDF)

S2 Fig Protocol for collecting data from trophy hunted lion *Panthera leo* and leopard to assist the sexing and aging of harvested individuals. (TIF)

S1 Table GLM parameter estimates using only radio-collared leopards with ≥30 telemetry locations. Model input and structure remained constant for comparison to our model using ≥10 telemetry locations.
(DOCX)

S1 Dataset Intrinsic susceptibility. Data used for the examination of intrinsic leopard susceptibility.
(CSV)

S2 Dataset Extrinsic susceptibility hunter attributes. Data used for the examination of hunter attributes as part of extrinsic leopard susceptibility.
(CSV)

S3 Dataset Extrinsic susceptibility leopard attributes. Data used for the examination of extrinsic susceptibility of leopards to trophy hunting.
(CSV)

S4 Dataset Requested code for analysis of leopard susceptibility data. Critical code for running leopard susceptibility models in R.
(DOCX)
