# Supplementary material for: Who Bites the Bullet First? The Susceptibility of Leopards Panthera pardus to Trophy Hunting
Source: PLoS One. 2015 Apr 10;10(4):e0123100. doi: 10.1371/journal.pone.0123100 (PMC4393264; doi:10.1371/journal.pone.0123100)

TROPHY HUNTER SELECTIVITY SURVEY

Below are photographs of known-age male & female leopards. Please indicate whether you would hunt the leopard on:

A) the 1st day of a 14-day safari,
B) the 7th day of a 14-day safari,
C) the 14th day of a 14-day safari, or
D) at no stage during the safari (i.e. you would not hunt the leopard).

(1)

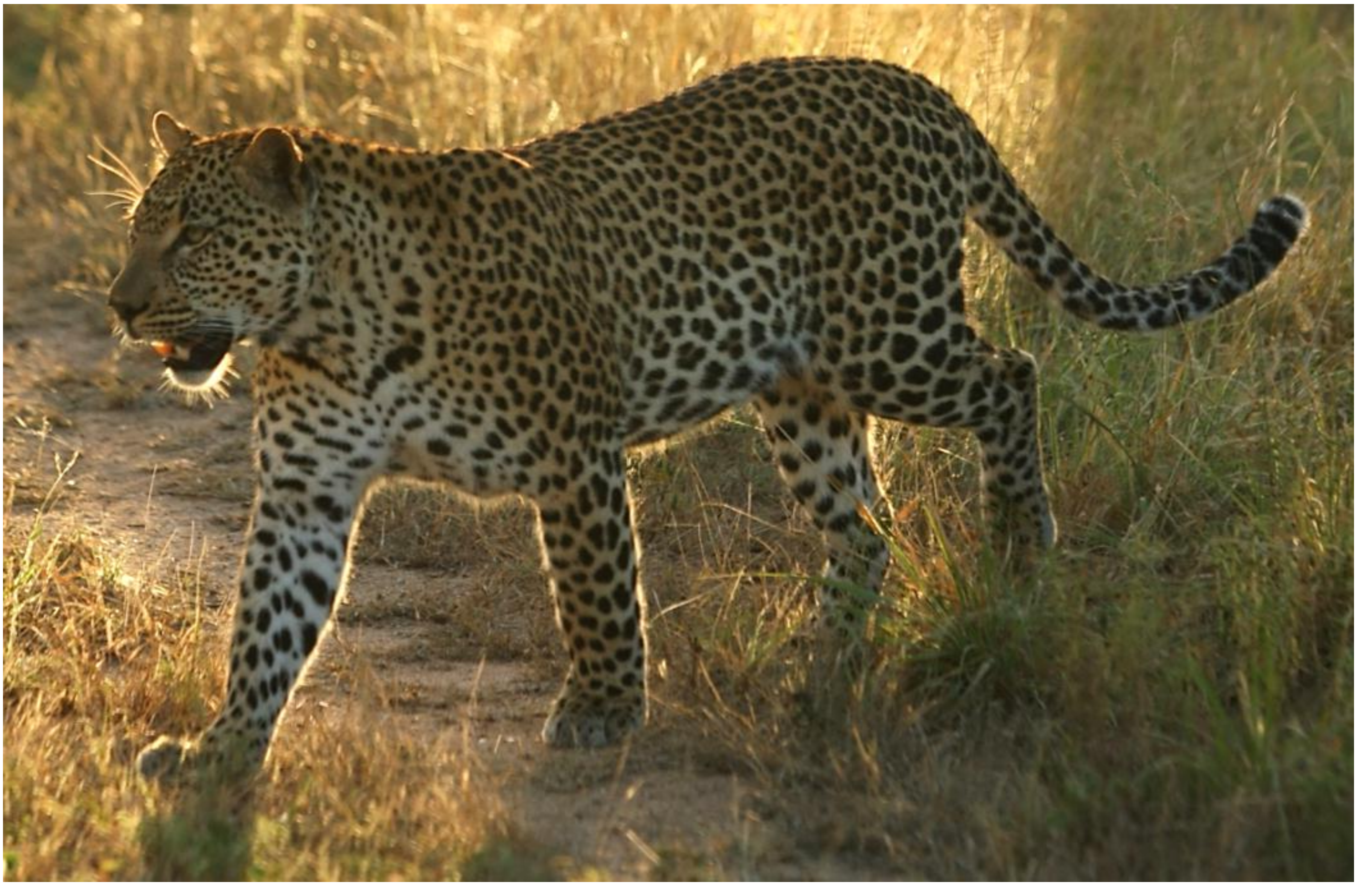

(2)

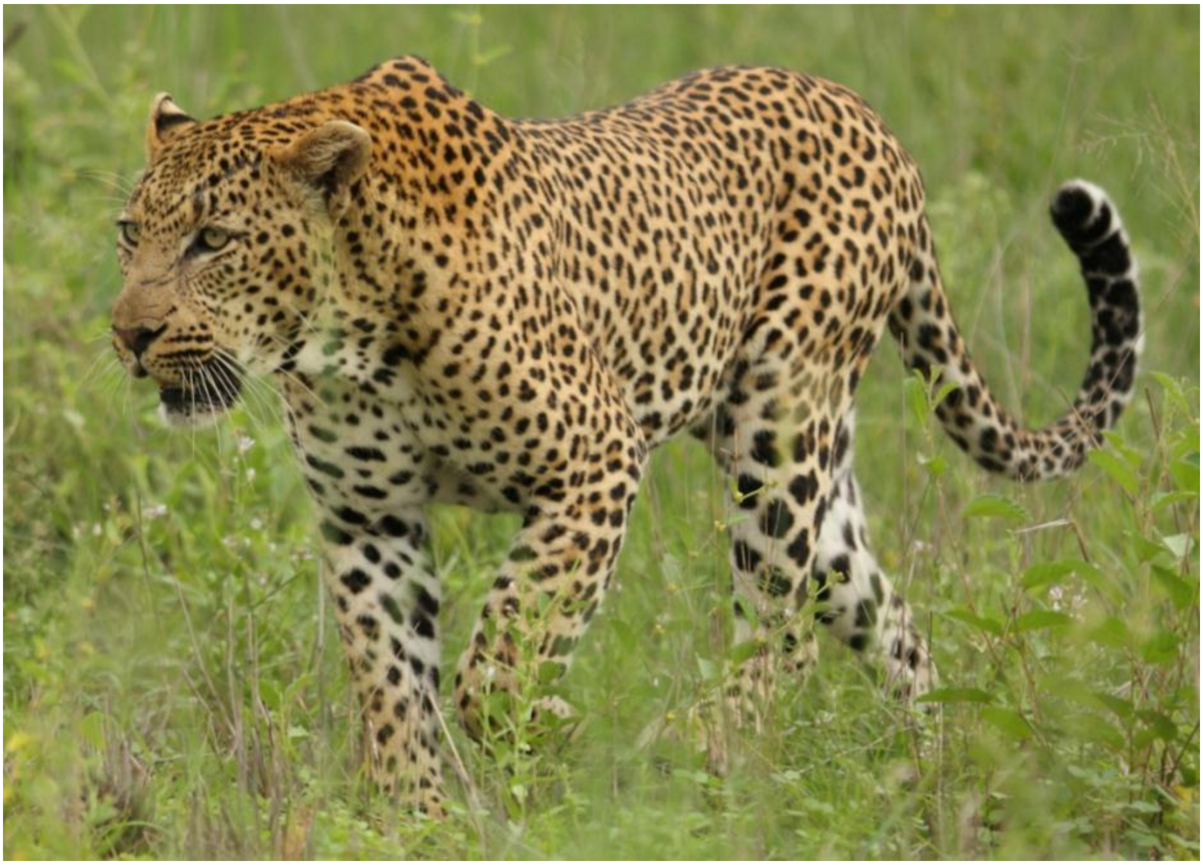

(3)

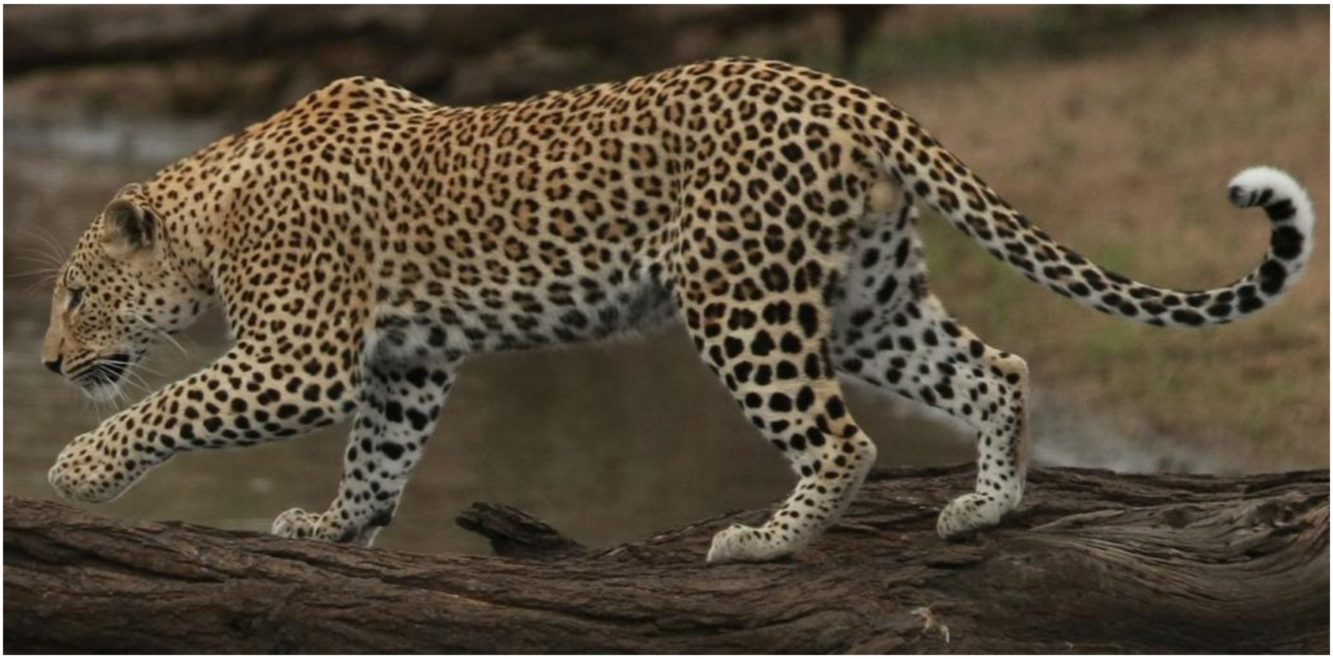

(4)

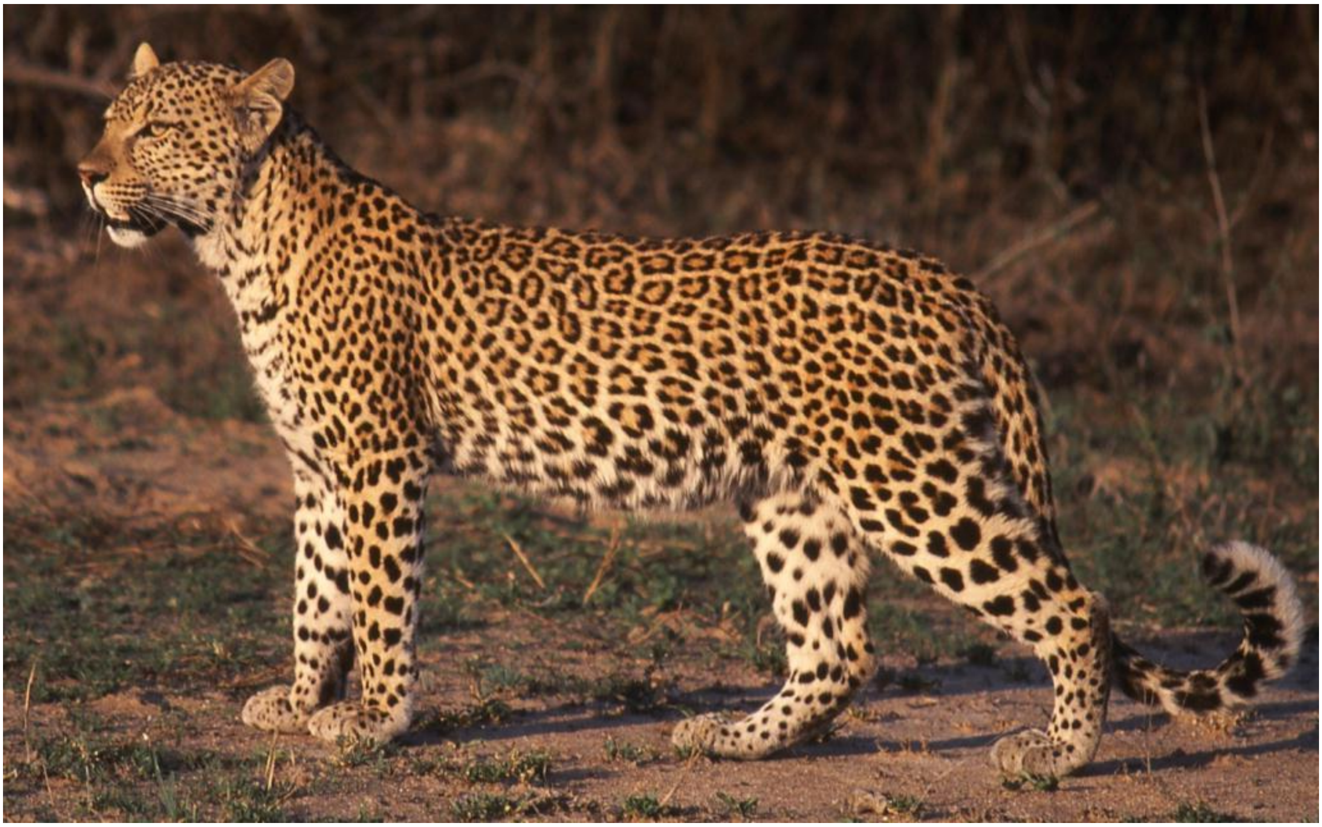

(5)

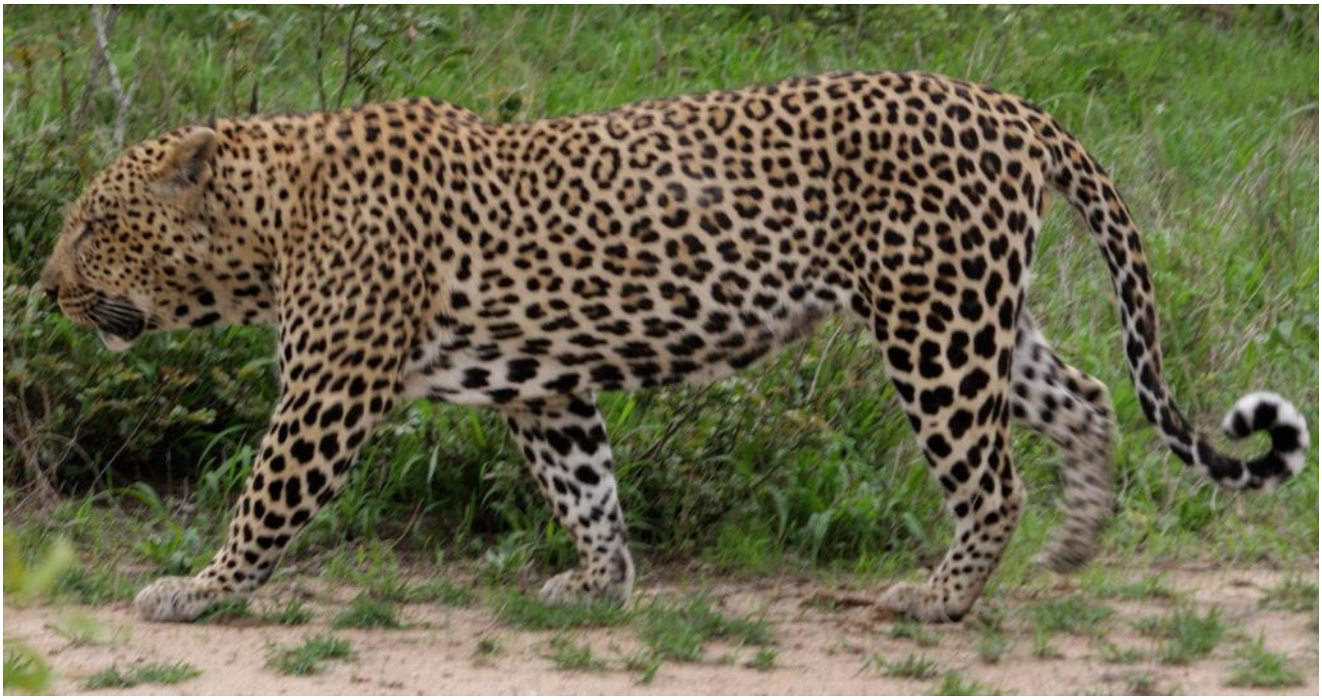

(6)

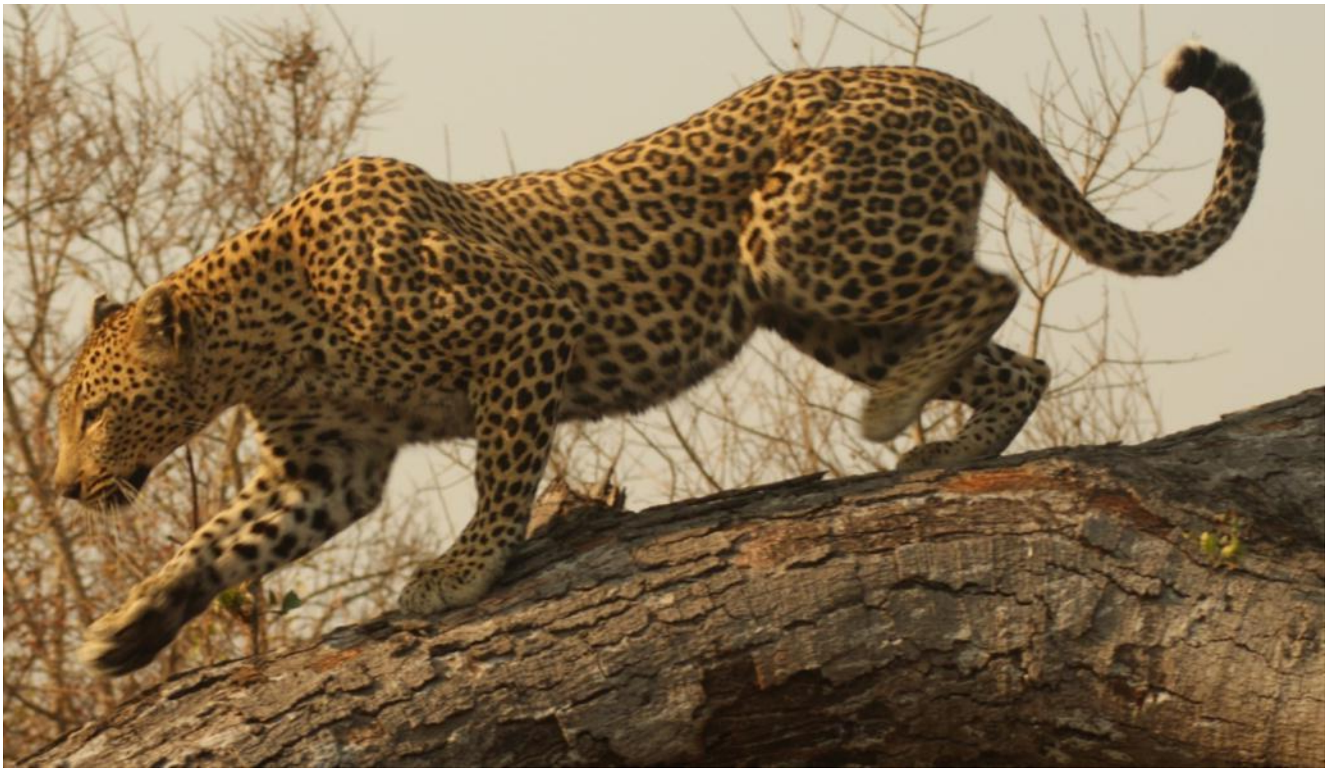

(7)

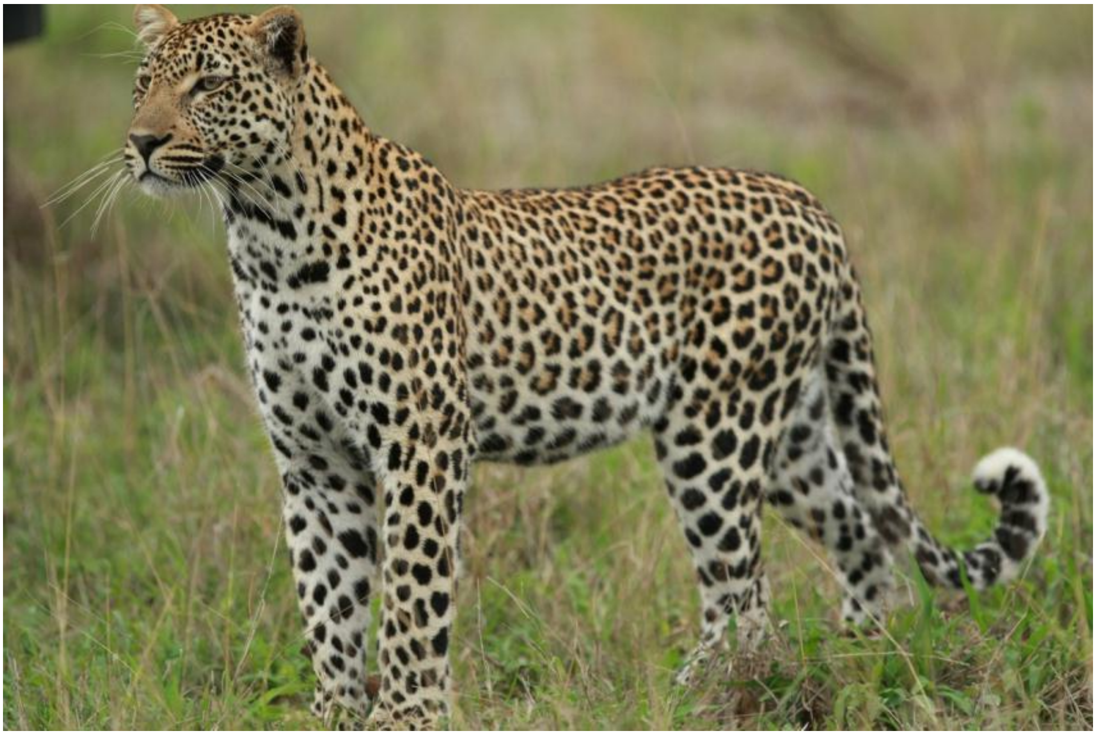

(8)

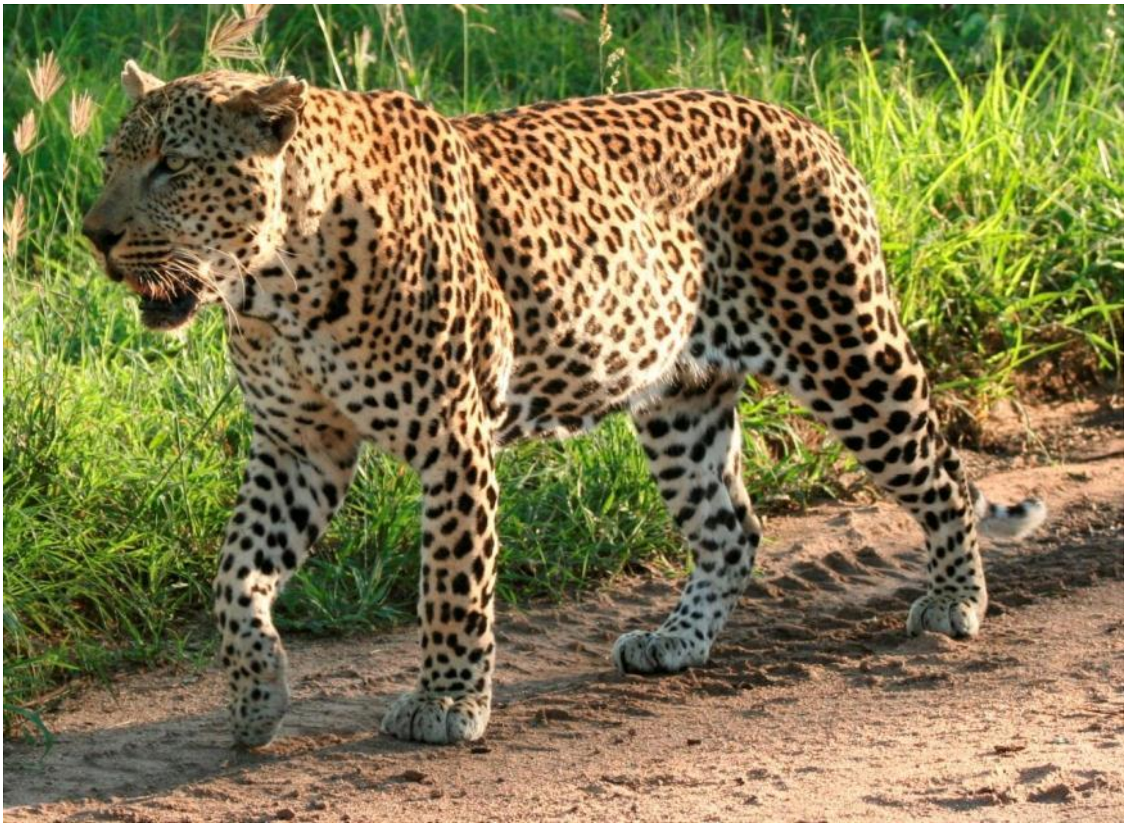

(9)

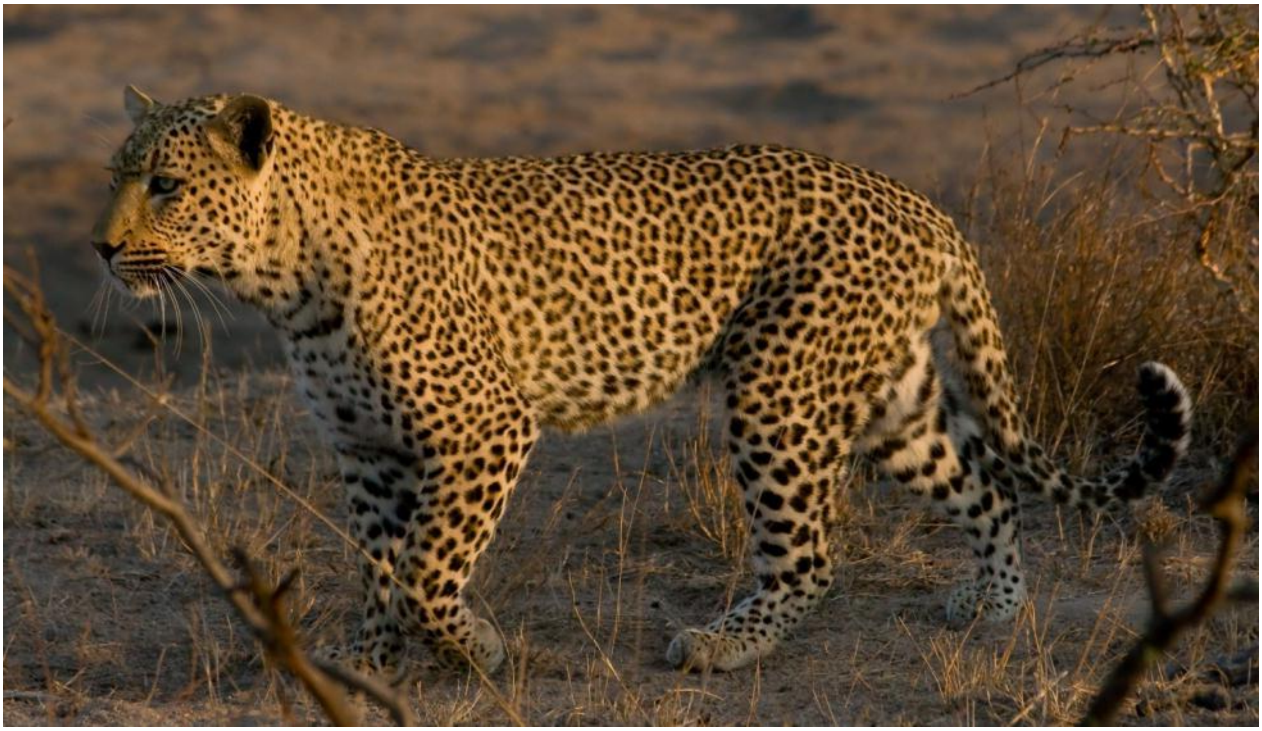

(10)

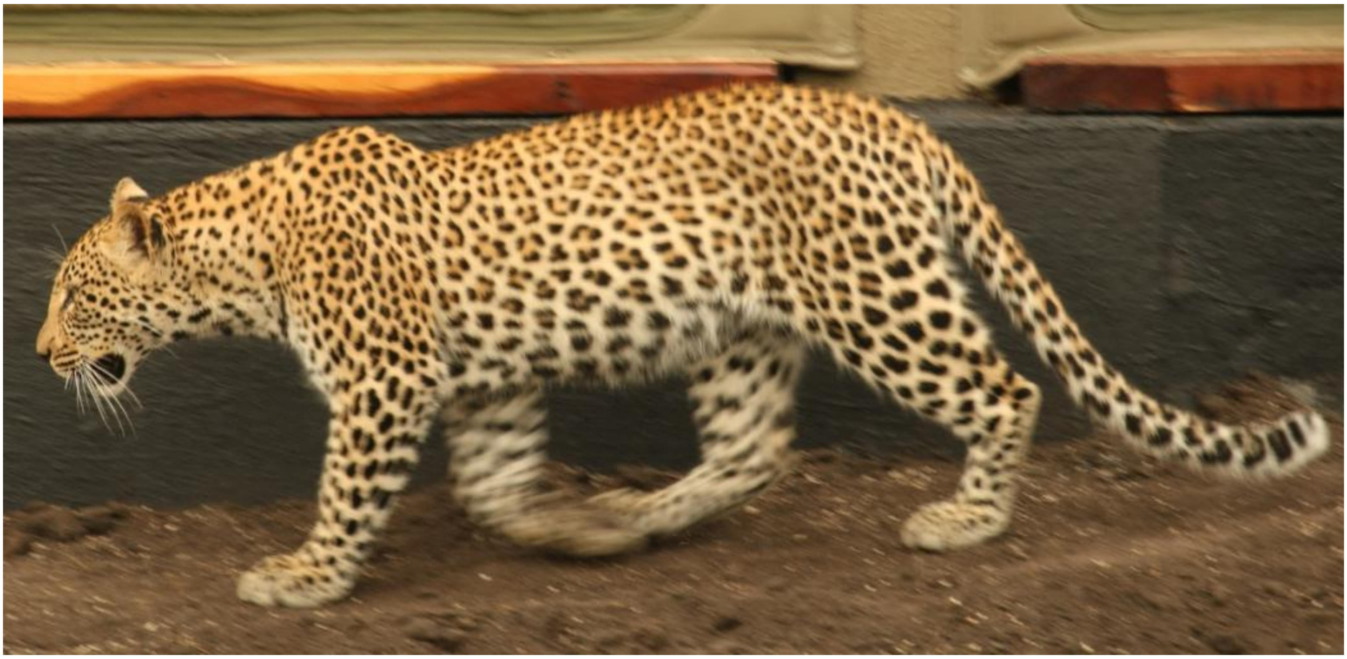

(11)

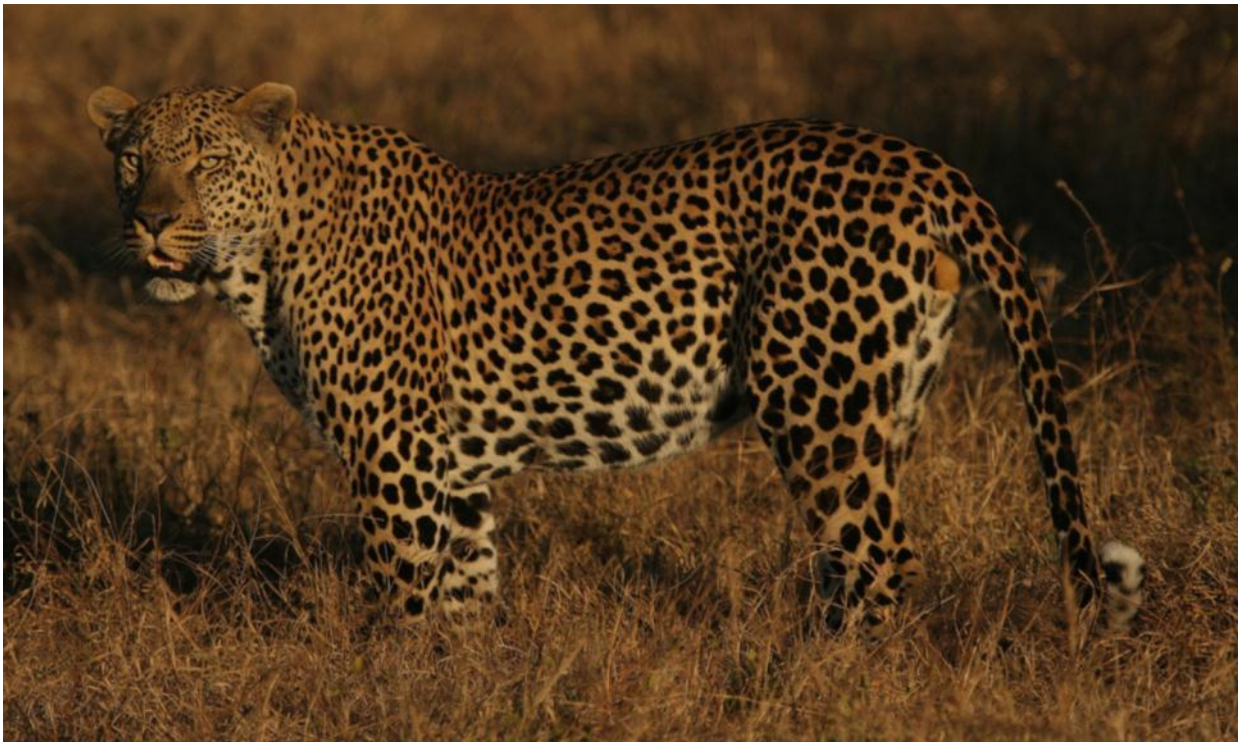

(12)

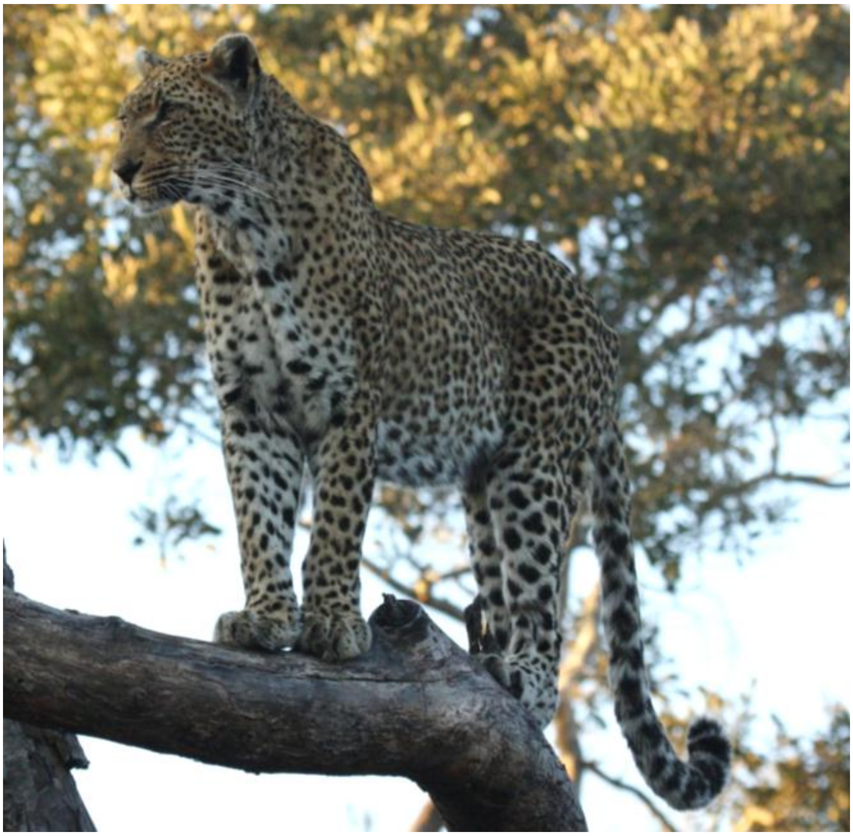

(13)

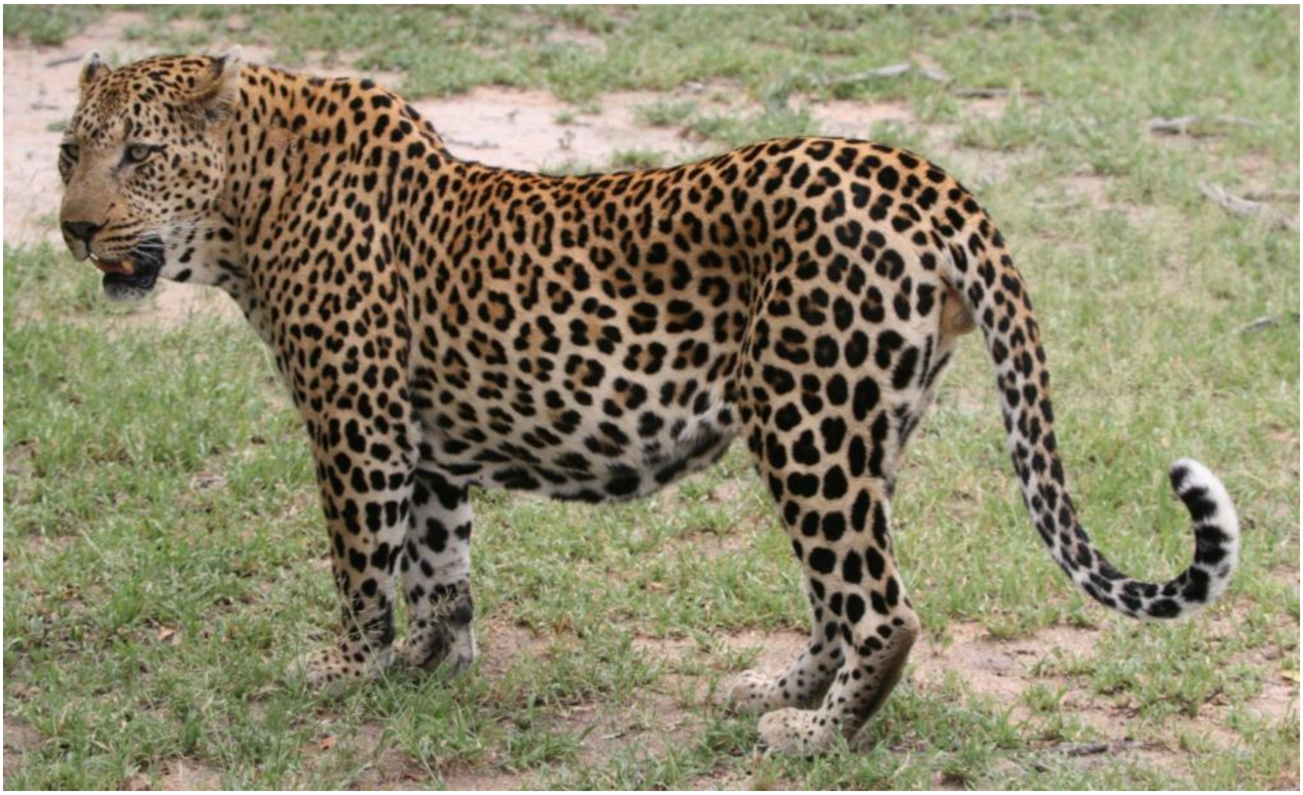

(14)

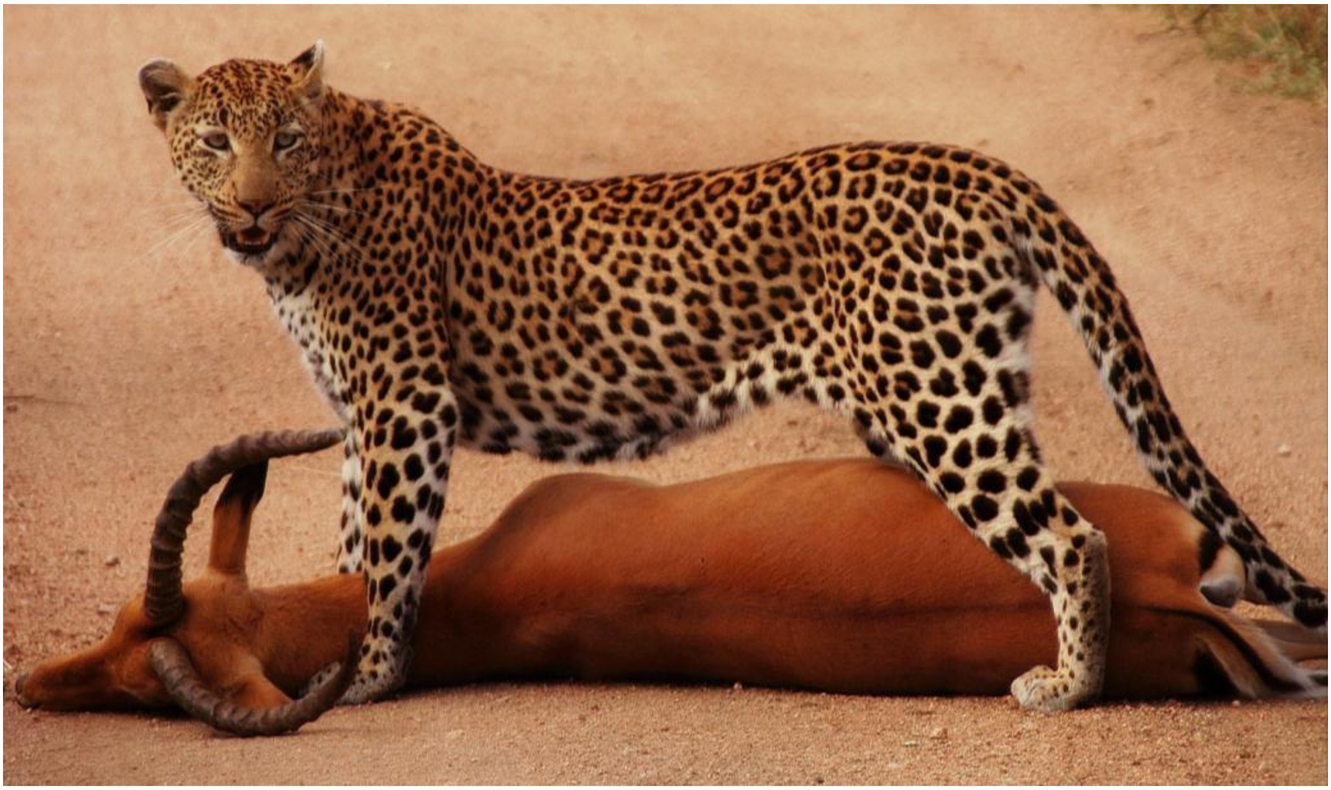

(15)

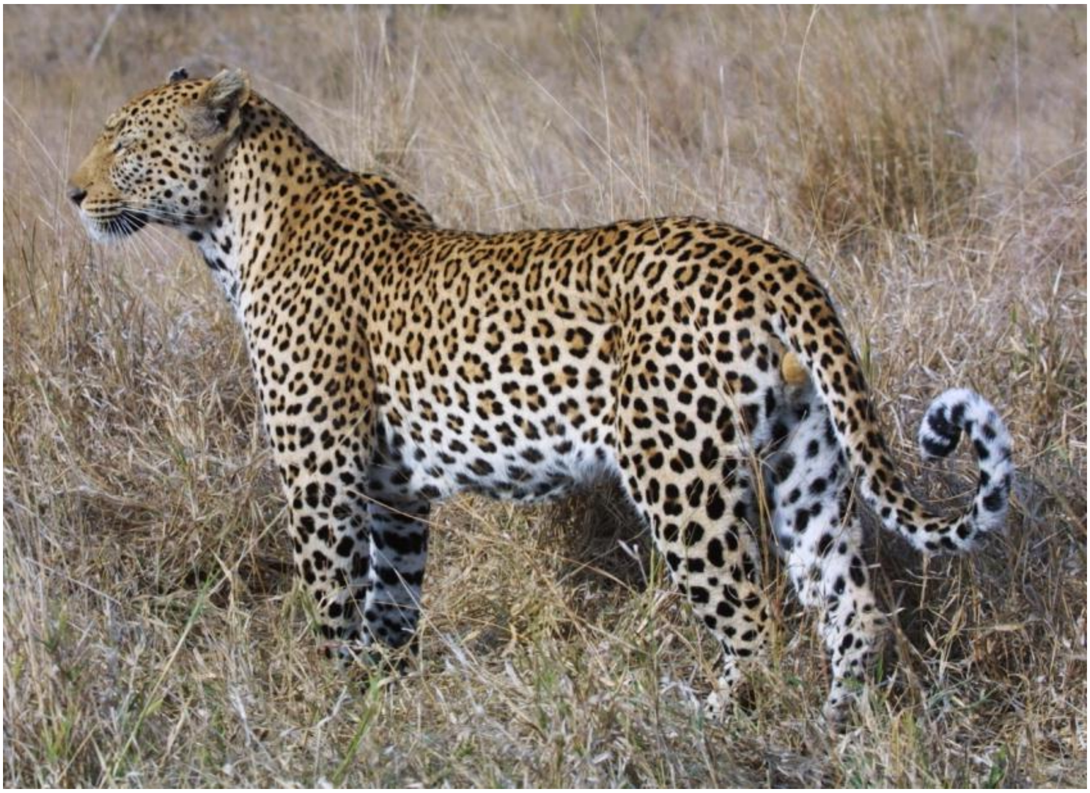

(16)

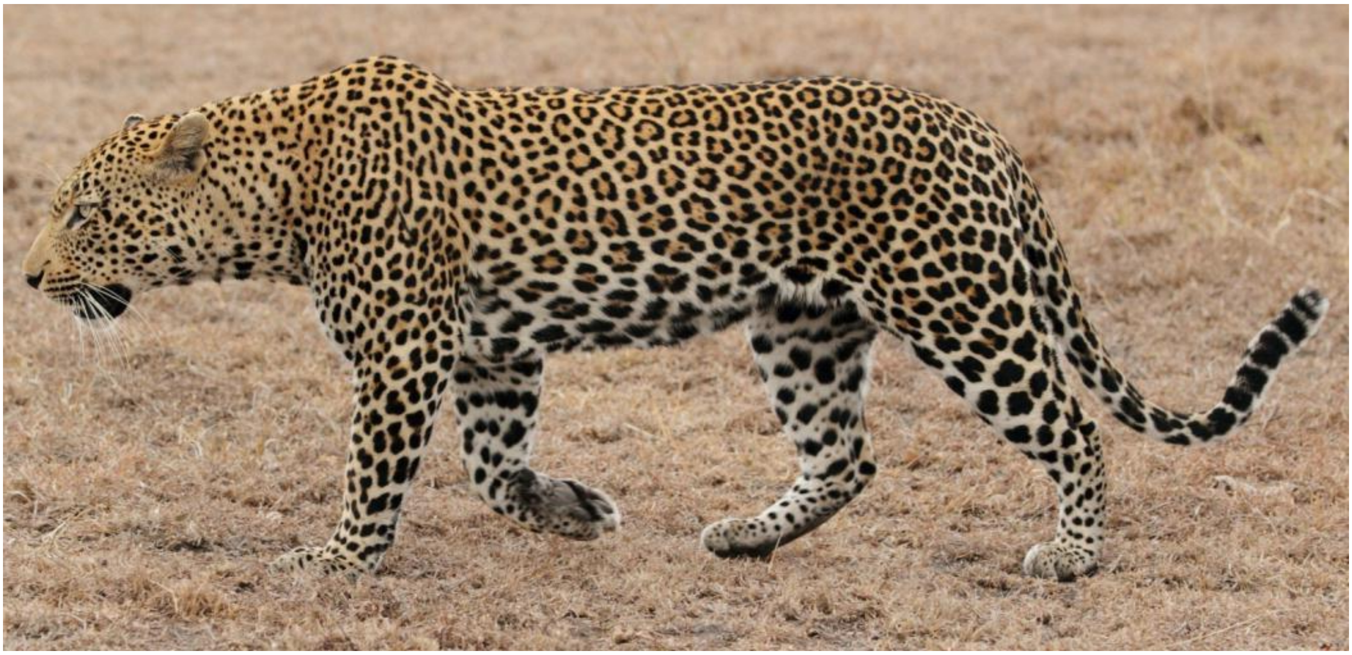

(17)

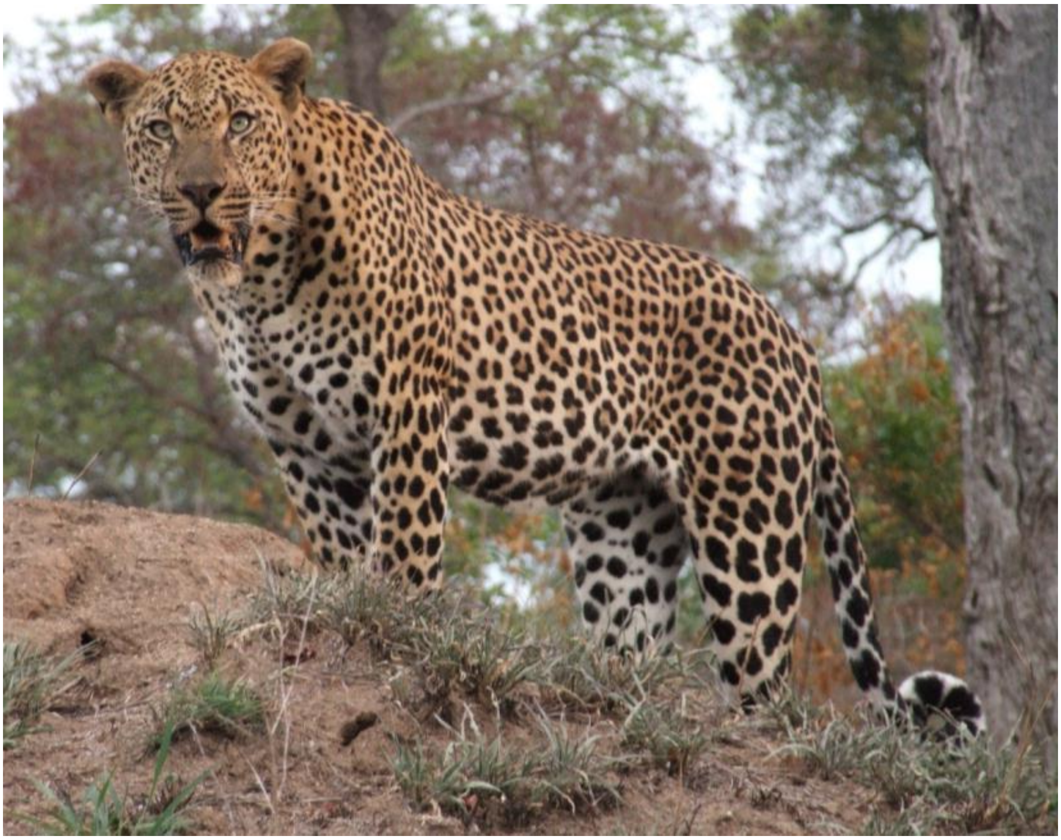

(18)

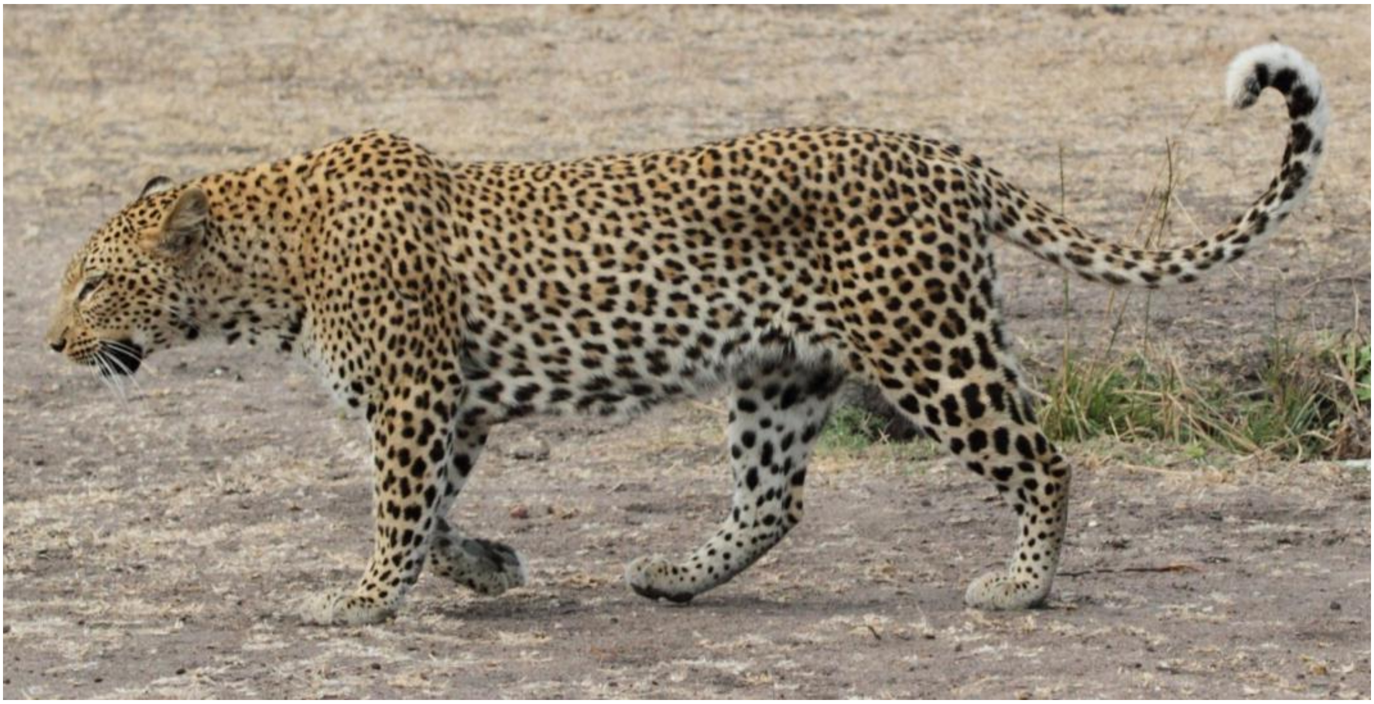

(19)

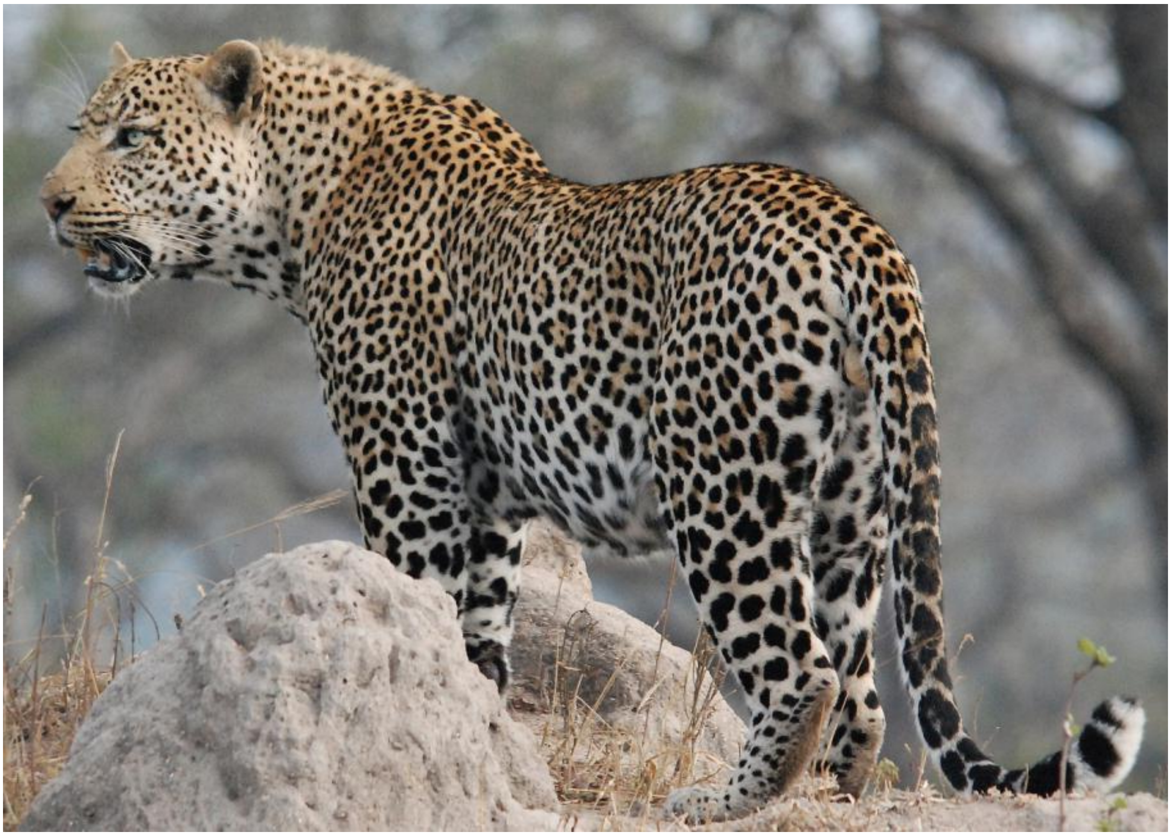

(20)

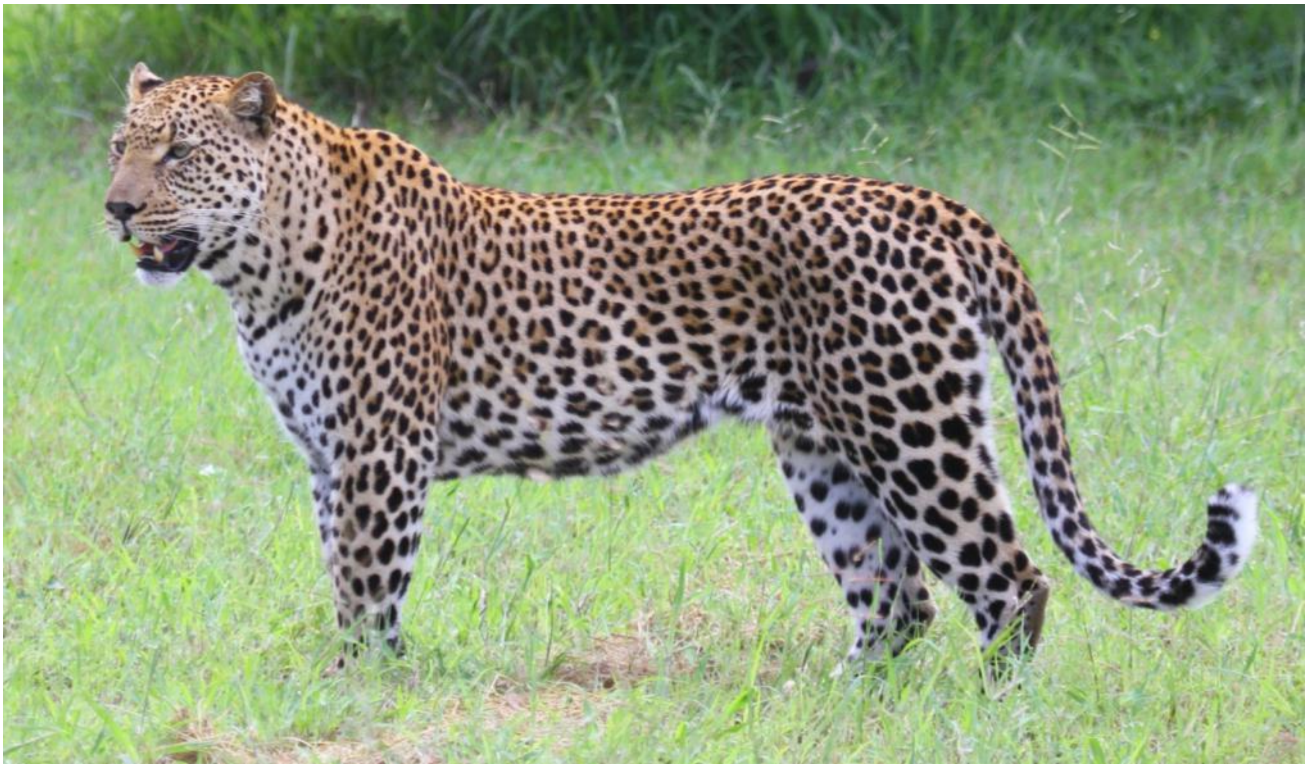

(21)

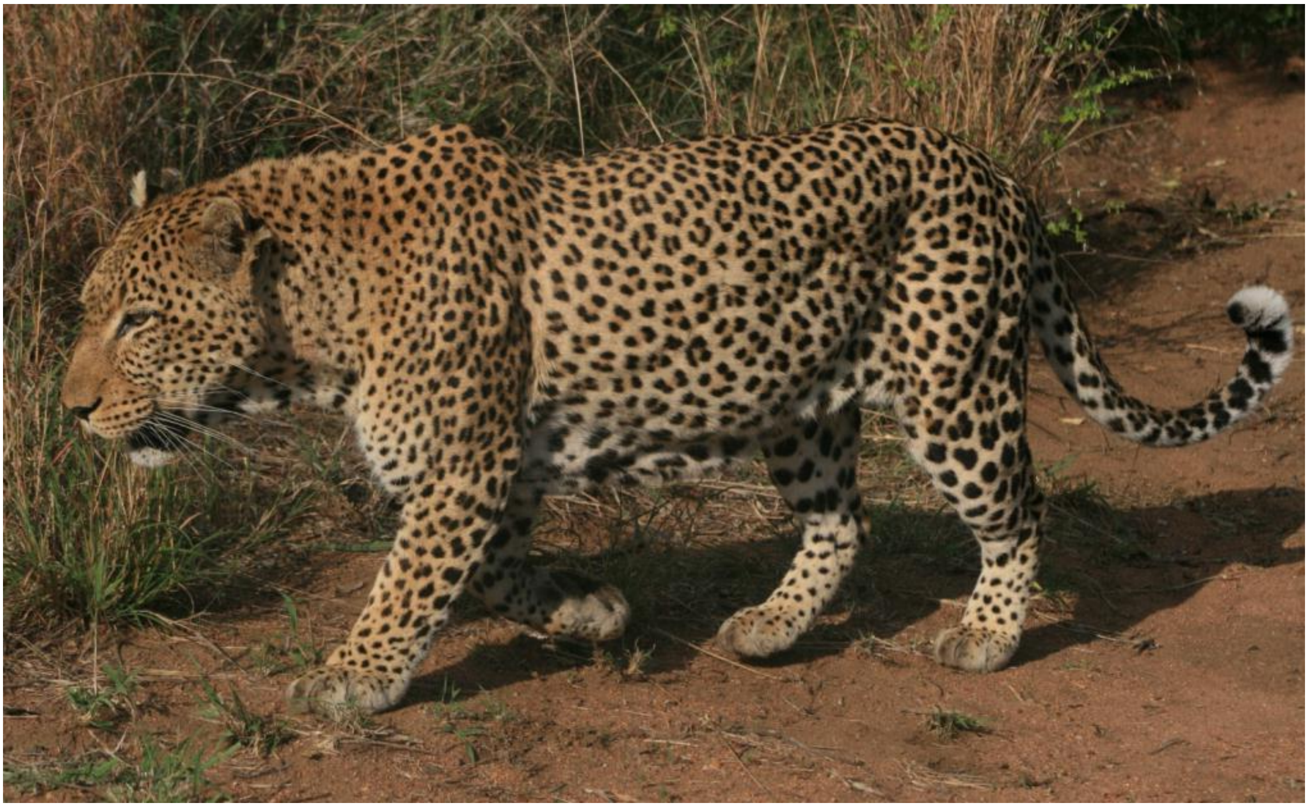

(22)

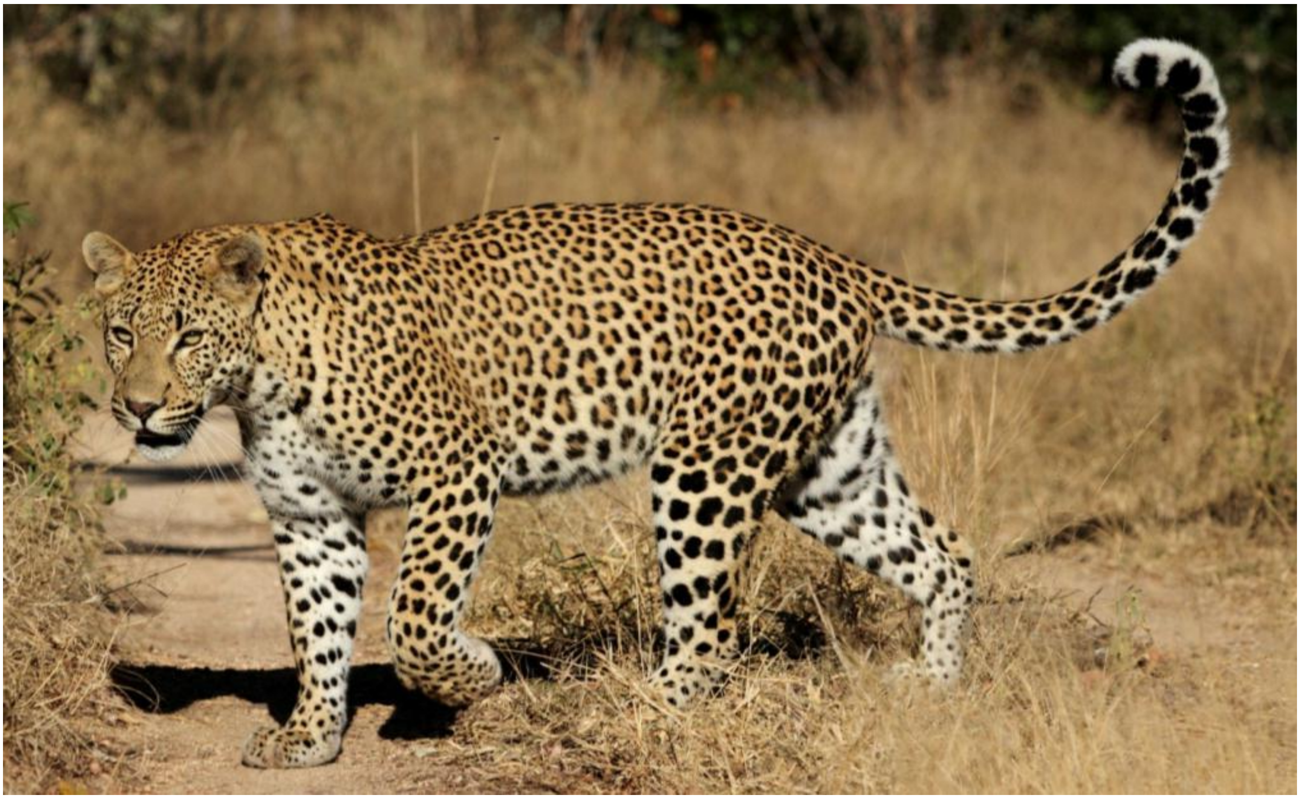

(23)

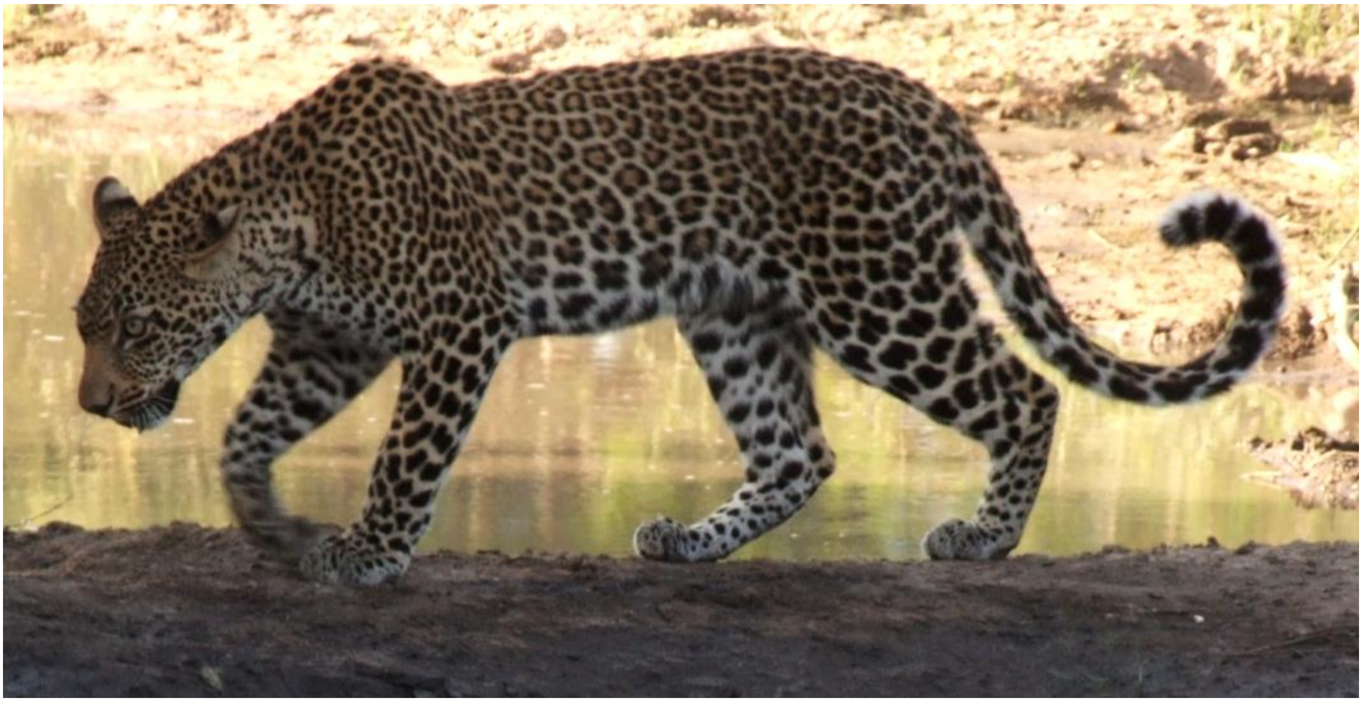

(24)

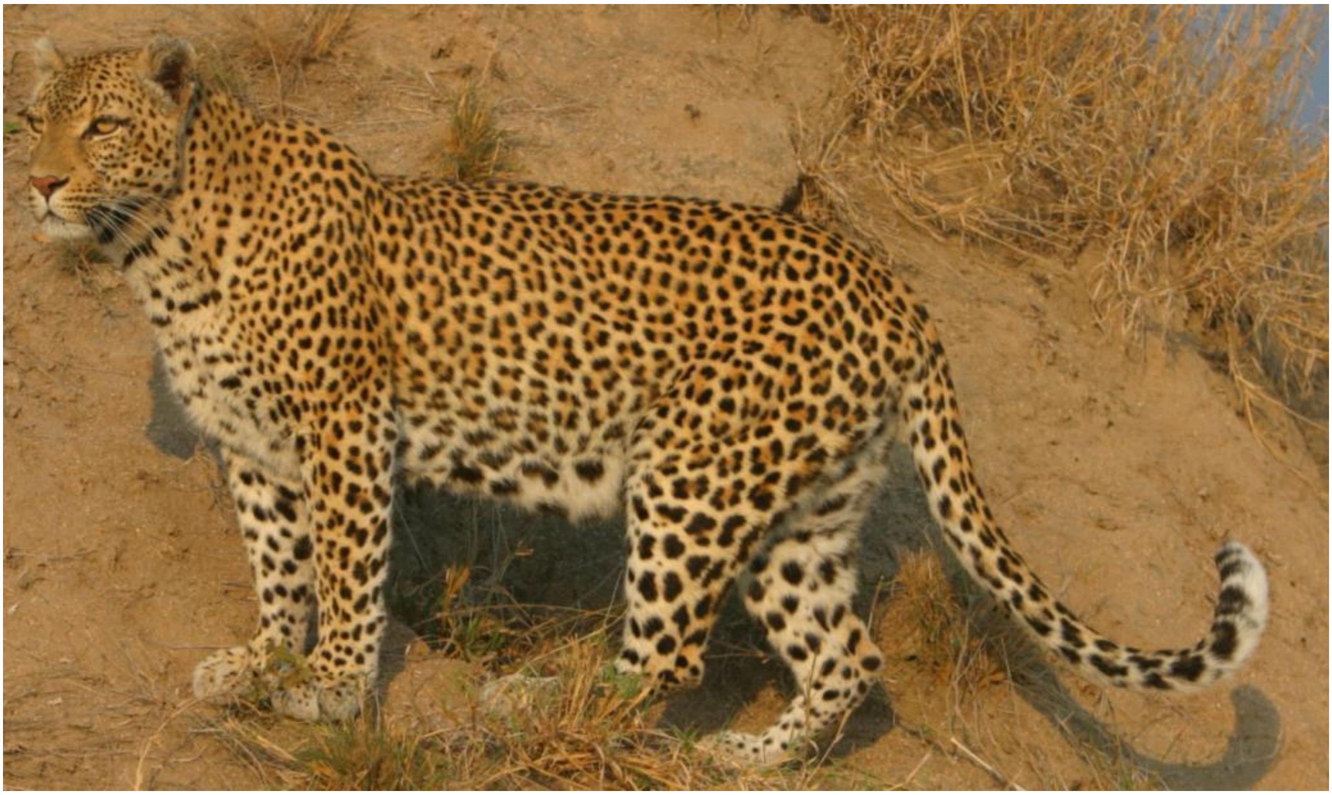

(25)

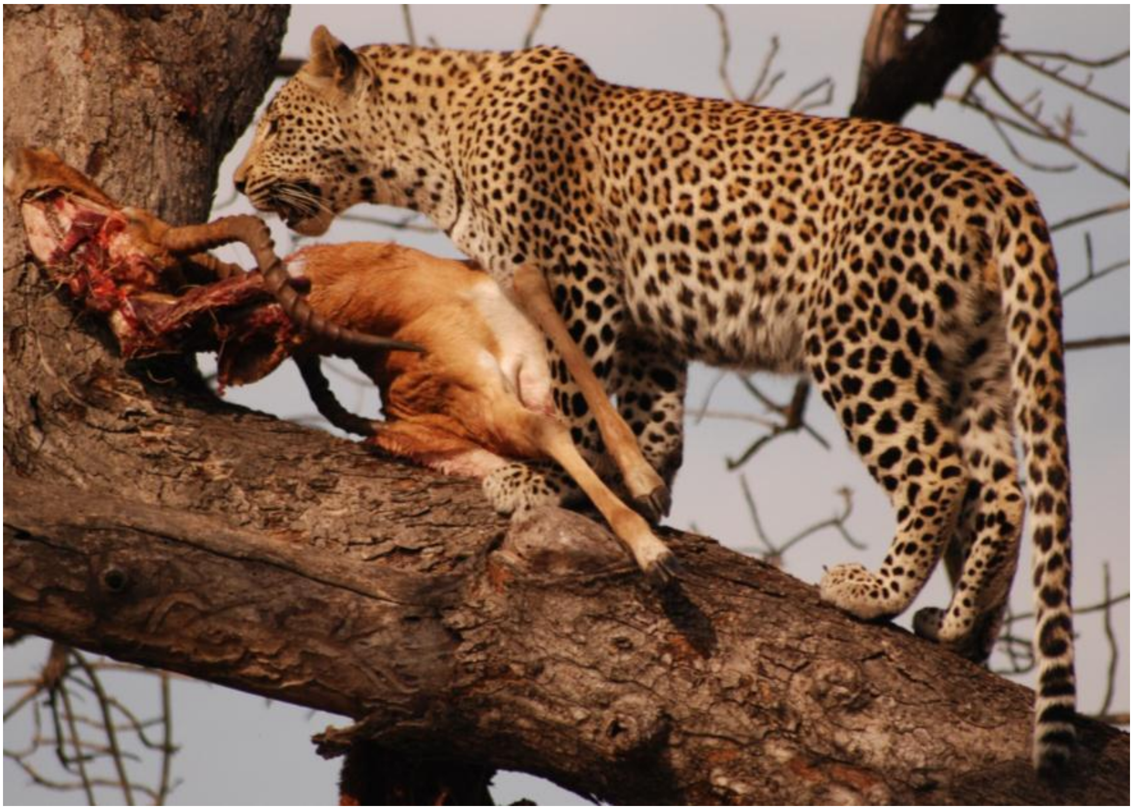

Supplement: S1 Fig — Respondents were asked whether they would choose to shoot a leopard, and on which day during a 14-day hunting safari they would make this decision: (a) 3 = willing to shoot on the first day of a 14-day safari; (b) 2 = willing to shoot on the seventh day of a 14-day safari; (c) 1 = willing to shoot on the fourteenth day of a 14-day safari (d) 0 = unwilling to shoot the leopard at any stage of the hunt. All photographs are of known sex and age leopards from the Sabi Sand Game Reserve, South Africa. (PDF) [file pone.0123100.s001.pdf]
