# Supplementary material for: Who Bites the Bullet First? The Susceptibility of Leopards Panthera pardus to Trophy Hunting
Source: PLoS One. 2015 Apr 10;10(4):e0123100. doi: 10.1371/journal.pone.0123100 (PMC4393264; doi:10.1371/journal.pone.0123100)
Supplement: S1 Table — Model input and structure remained constant for comparison to our model using ≥10 telemetry locations. (DOCX) [file pone.0123100.s003.docx]

| **Coefficients** | **Estimate** | **2.5% CI** | **97.5% CI** | **Exponentiated estimate** | **Std.error** | **z-value** | **Pr (>\|z\|)** |
| --- | --- | --- | --- | --- | --- | --- | --- |
| Intercept (Adult females) | -3.41 | -3.94 | -2.87 | 3.3 | 0.27 | -12.56 | <0.005 |
| Adult males | 0.26 | -0.7 | 1.23 | 4.29 | 0.49 | 0.52 | 0.6 |
| Sub-adults | -1.88 | -4.85 | -0.06 | 0.5 | 1.11 | -1.69 | 0.09 |

Residual deviance: 17.99 on 13 d.f

AIC: 68.63

Theta: 2.93
