# Supplementary material for: Who Bites the Bullet First? The Susceptibility of Leopards Panthera pardus to Trophy Hunting
Source: PLoS One. 2015 Apr 10;10(4):e0123100. doi: 10.1371/journal.pone.0123100 (PMC4393264; doi:10.1371/journal.pone.0123100)
Supplement: S4 Dataset — Critical code for running leopard susceptibility models in R. (DOCX) [file pone.0123100.s007.docx]

**Requested code for analysis of leopard susceptibility data:**

This first section of code is for the analysis of leopard susceptibility to hunting blinds as represented by our camera-traps, what we term ecological susceptibility.

You’ll need the following packages to run the analysis and if you would like to go on and plot some of the data:

Install.packages(foreign)

Install.packages(ggplot2)

Install.packages(MASS)

Go ahead and plot the four candidate models, using the negative binomial fit for the data:

Model 1 = glm.nb (Events ~ as.factor(Year) + offset(log(Time.in)))

Model 2 = glm.nb (Events ~ as.factor(Cohort) + as.factor(Year) + offset(log(Time.in)))

Model 3 = glm.nb (Events ~ as.factor(Cohort) + offset(log(Time.in)))

Model 4 = glm.nb (Events ~ as.factor(Cohort))

Compare th models using Aikaike’s Information Criterion:

AIC(Model1,Model2,Model3,Model4)

Model 3 records the lowest score and has the lowest number of parameters:

Summary(Model3)

It’s worth taking a look at the over dispersion of a Poisson fit:

Model3pois = glm(formula = Events ~ as.factor(Cohort) + offset(log(Time.in)))

Now let’s have a look at the over dispersion using a quassipoisson fit:

Model3quas = glm(formula = Events ~ as.factor(Cohort) + offset(log(Time.in)), family = quasipoisson)

Just to be certain compare a Poisson model to a negative binomial fit:

pchisq(2 * (logLik(Model3) - logLik(Model3pois)), df = 1, lower.tail = FALSE)

Generate some confidence intervals:

(est <- cbind(Estimate = coef(Model3), confint(Model3)))

This second section of code is for the analysis of leopard susceptibility to hunters, as revealed by our questionnaire sheet.

The first section explores leopard attributes on score (average score as predicted by Cohort).

Leopardmodel = glm(formula = Score ~ Age.Sex.class)

Summary(Leopardmodel)

The second section explores the attributes of hunters on the average score they recorded for the test.

Huntermodel = glm(formula = Average.score ~ Years + Hunts.guided + Countries)

Summary(Huntermodel)
